# Compositionally Sequenced Interfacial Layers for High‐Energy Li‐Metal Batteries

**DOI:** 10.1002/advs.202310094

**Published:** 2024-02-26

**Authors:** Jeong‐A Lee, Saehun Kim, Yoonhan Cho, Seong Hyeon Kweon, Haneul Kang, Jeong Hwan Byun, Eunji Kwon, Samuel Seo, Wonkeun Kim, Kyoung Han Ryu, Sang Kyu Kwak, Seungbum Hong, Nam‐Soon Choi

**Affiliations:** ^1^ Department of Chemical and Biomolecular Engineering Korea Advanced Institute of Science and Technology (KAIST) 291 Daehak‐ro, Yuseong‐gu Daejeon 34141 Republic of Korea; ^2^ Department of Materials Science and Engineering Korea Advanced Institute of Science and Technology (KAIST) 291 Daehak‐ro, Yuseong‐gu Daejeon 34141 Republic of Korea; ^3^ School of Energy and Chemical Engineering Ulsan National Institute of Science and Technology (UNIST) 50 UNIST‐gil Ulsan 44919 Republic of Korea; ^4^ CTO Advanced Battery Development Hyundai motor company 37 Cheoldobangmulgwan‐ro Uiwang‐si Gyeonggi‐do 16082 Republic of Korea; ^5^ Department of Chemical and Biological Engineering Korea University 145 Anam‐ro, Seongbuk‐gu Seoul 02841 Republic of Korea

**Keywords:** cathode‐electrolyte interphase, electrolyte additives, Li‐metal batteries, Ni‐rich cathodes, solid electrolyte interphase

## Abstract

Electrolyte additives with multiple functions enable the interfacial engineering of Li‐metal batteries (LMBs). Owing to their unique reduction behavior, additives exhibit a high potential for electrode surface modification that increases the reversibility of Li‐metal anodes by enabling the development of a hierarchical solid electrolyte interphase (SEI). This study confirms that an adequately designed SEI facilitates the homogeneous supply of Li^+^, nonlocalized Li deposition, and low electrolyte degradation in LMBs while enduring the volume fluctuation of Li‐metal anodes on cycling. An in‐depth analysis of interfacial engineering mechanisms reveals that multilayered SEI structures comprising mechanically robust LiF‐rich species, electron‐rich P–O species, and elastic polymeric species enabled the stable charge and discharge of LMBs. The polymeric outer SEI layer in the as‐fabricated multilayered SEI could accommodate the volume fluctuation of Li‐metal anodes, significantly enhancing the cycling stability Li||LiNi_0.8_Co_0.1_Mn_0.1_O_2_ full cells with an electrolyte amount of 3.6 g Ah^−1^ and an areal capacity of 3.2 mAh cm^−2^. Therefore, this study confirms the ability of interfacial layers formed by electrolyte additives and fluorinated solvents to advance the performance of LMBs and can open new frontiers in the fabrication of high‐performance LMBs through electrolyte‐formulation engineering.

## Graphical abstract

The unique reduction behavior of additives enables the creation of a multilayered solid electrolyte interphase (SEI) comprising mechanically robust LiF, polar P–O, and elastic polymeric species on a Li‐metal anode. Adequately designed SEI layers lead to uniform Li deposition and suppression of electrolyte degradation in Li‐metal batteries, while enduring huge volume fluctuation of Li‐metal on cycling.

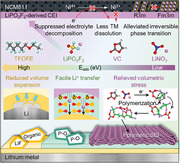

## 1 Introduction

With the global shift from vehicles with traditional internal combustion engines to electric vehicles to meet global climate targets, Li‐metal batteries (LMBs) comprising high‐capacity electrode materials with Li‐metal have rapidly gained prominence.^[^ ^1^, ^2^, ^3^ ^]^ Despite exhibiting a high energy density per unit weight and low oxidation‐reduction potential (−3.04 V vs SHE, standard hydrogen electrode), the high reactivity of Li‐metal induces the uncontrolled deterioration of the electrolyte components in LMBs; this strengthens the blocking character of the solid electrolyte interphase (SEI) and reduces the Coulombic efficiency (CE) of the system, thereby reducing the lifespan of the device.^[^ ^4^, ^5^, ^6^, ^7^, ^8^, ^9^, ^10^, ^11^, ^12^, ^13^, ^14^ ^]^ Mechanically fragile organic species are unable to manipulate the unwanted reactions of the Li‐metal anode with electrolyte in LMBs owing to the high probability of SEI cracking upon repetitive Li deposition and stripping.^[^ ^15^, ^16^, ^17^, ^18^ ^]^ The infiltration of the electrolyte to the vicinity of the Li‐metal anode through cracks in the SEI causes sustained electrolyte decomposition, resulting in rapid electrolyte consumption. Consequently, several studies have endeavored to overcome the low mechanical fragility and compositional inhomogeneity of SEIs. Low reactivity was observed between the solvent molecules and Li‐metal electrode in electrolytes with a high salt concentration owing to the presence of inorganic SEI species formed by the decomposition of salt.^[^ ^19^, ^20^, ^21^, ^22^, ^23^, ^24^ ^]^ Additionally, localized highly concentrated electrolytes (LHCEs), which are achieved by incorporating fluorinated solvents into highly concentrated electrolytes (HCEs), overcome the intrinsic limitations of HCEs such as high viscosity and poor wettability.^[^ ^25^, ^26^, ^27^, ^28^ ^]^ LHCEs increase the proportion of cation‐anion aggregate species in the solvation structure of the electrolyte and aid in the construction of an anion‐driven SEI on the Li‐metal anode (Figure S1, Supporting Information).

Furthermore, despite the low concentration of lithium salts in LHCEs, the proportion of cation‐anion aggregates in LHCEs containing an excess of diluents is higher than that of the aggregates present in electrolytes with high salt concentrations (Figure S2, Supporting Information). Nevertheless, the practical utilization of LHCEs is limited by the high price of Li salts and fluorinated solvents. Notably, the introduction of fluorinated solvents as diluents increases the density of LHCEs; therefore, considering the inclusion of a fixed weight of an electrolyte, the LHCE volume that is injected into an LMB is smaller than that of a low‐density electrolyte. Further, insufficient wetting of the electrolyte in the electrode hinders facile Li^+^‐ion transport within the electrode (Table S1 and Figure S3, Supporting Information).^[^ ^27^, ^29^, ^30^, ^31^, ^32^ ^]^ Consequently, enabling the stable operation of LMBs under a lean‐electrolyte system is a vital aspect of fabricating high‐energy‐density LMB systems.^[^ ^33^, ^34^, ^35^, ^36^ ^]^ Notably, Li‐metal anodes without adequate SEI layers exhibit poor electrochemical reversibility. SEI engineering can be used to overcome this limitation and advance the electrochemical behavior of Li‐metal anodes. Among the different SEI‐synthesis strategies developed to date, electrolyte additives with different adsorption behaviors and reduction tendencies that undergo in situ electrochemical reactions at the Li‐metal anode are particularly suitable for the fabrication of stable SEIs.^[^ ^9^, ^37^, ^38^, ^39^, ^40^, ^41^ ^]^

This study reports a multilayer SEI comprising a polymeric outer layer, LiF‐enriched inner layer, and P–O‐species‐based middle layer synthesized by the sequential reduction of 1,1,2,2‐tetrafluoroethyl‐1H,1H,5H‐octafluoropentyl‐ether (TFOFE), lithium difluorophosphate (LiPO_2_F_2_), lithium nitrate (LiNO_3_), and vinylene carbonate (VC). LiF‐enriched inner SEIs driven by TFOFE exhibit several unique properties, including electronic insulation, high mechanical strength, and a wide electrochemical stability window.^[^ ^42^, ^43^ ^]^ The organic‐species‐containing inner layer and P–O‐species‐based middle layer of the as‐synthesized multilayer SEI facilitated Li^+^ transport across the SEI owing to their low compactness and electronegativity. Moreover, the construction of a polymeric outer SEI layer by VC and LiNO_3_ upon repetitive Li plating/stripping compensated for the insufficient mechanical integrity of the organic‐species‐containing inner SEI layer. The presence of insulating polymeric species in the upper regions of an SEI is expected to prevent electron conduction to regions that are in close proximity to the separator, guiding Li plating preferentially underneath the SEI, and dissipating the dynamic strain induced by repetitive Li plating/stripping, causing minimal SEI‐structure changes. In the as‐synthesized multilayer SEI, the reductive decomposition of VC formed polymeric species with a high elastic modulus such as poly(VC) that could adapt to the large volume fluctuation in Li‐metal anodes on cycling, while LiNO_3_ facilitated ion migration through the layer by generating ionically conductive Li_3_N species.^[^ ^44^, ^45^, ^46^, ^47^, ^48^ ^]^ The compositionally controlled multilayer SEI synthesized in this study enabled Li‐metal anodes to exhibit high electrochemical reversibility; moreover, it enhanced the cycling stability of Li||LiNi_0.8_Co_0.1_Mn_0.1_O_2_ (Li||NCM811) cells containing a LiPO_2_F_2_‐promoted cathode–electrolyte interphase (CEI) under the low E/C ratio of 3.6 mg mAh^−1^ and an areal capacity of 3.2 mAh cm^−2^.

## 2 Results and Discussion

### 2.1 Characterization of the Multilayer SEI Structure

**Figure 1:**
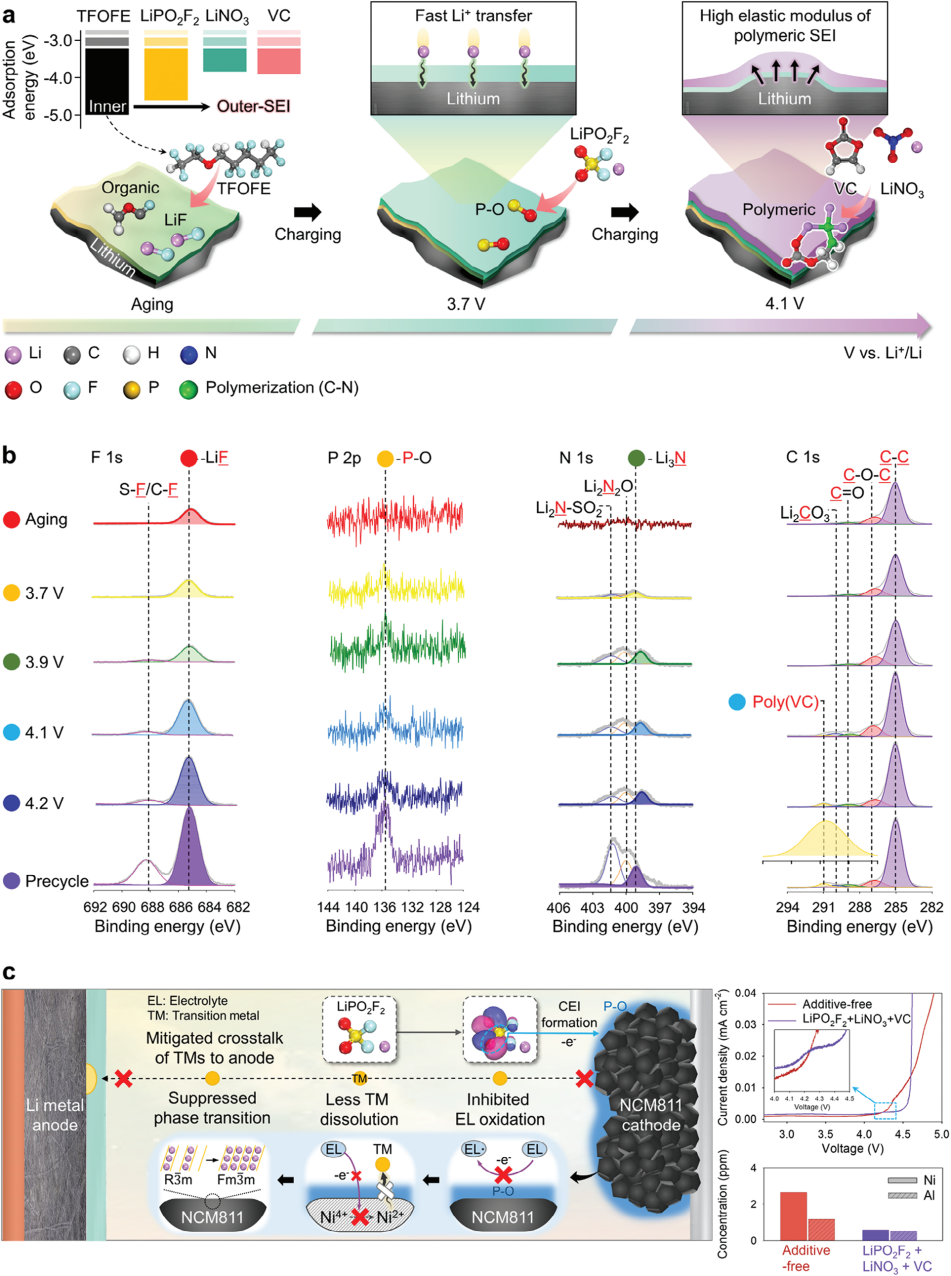
a) Schematics of the design and characterization of the newly proposed sequenced multilayer SEI on Li‐metal anodes. b) The XPS results of Li‐metal anodes in the LiPO_2_F_2_ + LiNO_3_ + VC electrolyte at different charge states. c) Effects of an LiPO_2_F_2_‐promoted polar CEI on NCM811 cathodes.

**Figure** **1**a shows the fabrication of the sequenced multilayer SEI proposed in this study on a Li‐metal anode in solutions containing a combination of three additives (LiPO_2_F_2_, LiNO_3_, and VC). Additionally, the figure highlights the influence of a LiPO_2_F_2_‐driven CEI on the synthesis of the multilayer SEI. The disparity in the adsorption energies of the additives and the TFOFE solvent enabled their sequential decomposition to generate a multilayer SEI on Li‐metal anodes. TFOFE primarily formed the LiF‐ and organic‐species‐based inner SEI layer, LiPO_2_F_2_ contributed to the construction of the middle SEI layer comprising species with the electronegative P and O atoms, while LiNO_3_ and VC induced the development of the outer SEI layer comprising ionically conductive polymeric species. In Figure 1b, the multilayer SEI structure was identified by X‐ray photoelectron spectroscopy (XPS). The co‐solvent TFOFE preferentially underwent the reaction to form LiF and several organic species at the Li‐metal surface during the aging process owing to its low adsorption energy. The decomposition of LiPO_2_F_2_, which started at 3.7 V versus Li/Li^+^, generated XPS peaks corresponding to species containing the P–O bond. Moreover, the XPS spectrum of the anode contained signals corresponding to LiNO_3_ and poly(VC) species at 3.9 and 4.1 V versus Li/Li^+^, respectively. Therefore, XPS confirmed that precycling Li‐metal anodes in different electrolyte solutions generated a multilayer‐structure SEI consisting of an inner layer comprising LiF and several organic species that mitigated anodic volume expansion, a P–O‐based middle layer that facilitated Li^+^ transfer, and a polymeric outer layer with a high elastic modulus on the anodes. Additionally, P–O‐based CEIs formed on NCM811 cathodes by LiPO_2_F_2_ alleviated the irreversible structural transition of the cathodes from the layer to the rock‐salt phase, reducing transition‐metal dissolution from the electrode and the electrolyte decomposition in the system (Figure 1c).

**Figure 2:**
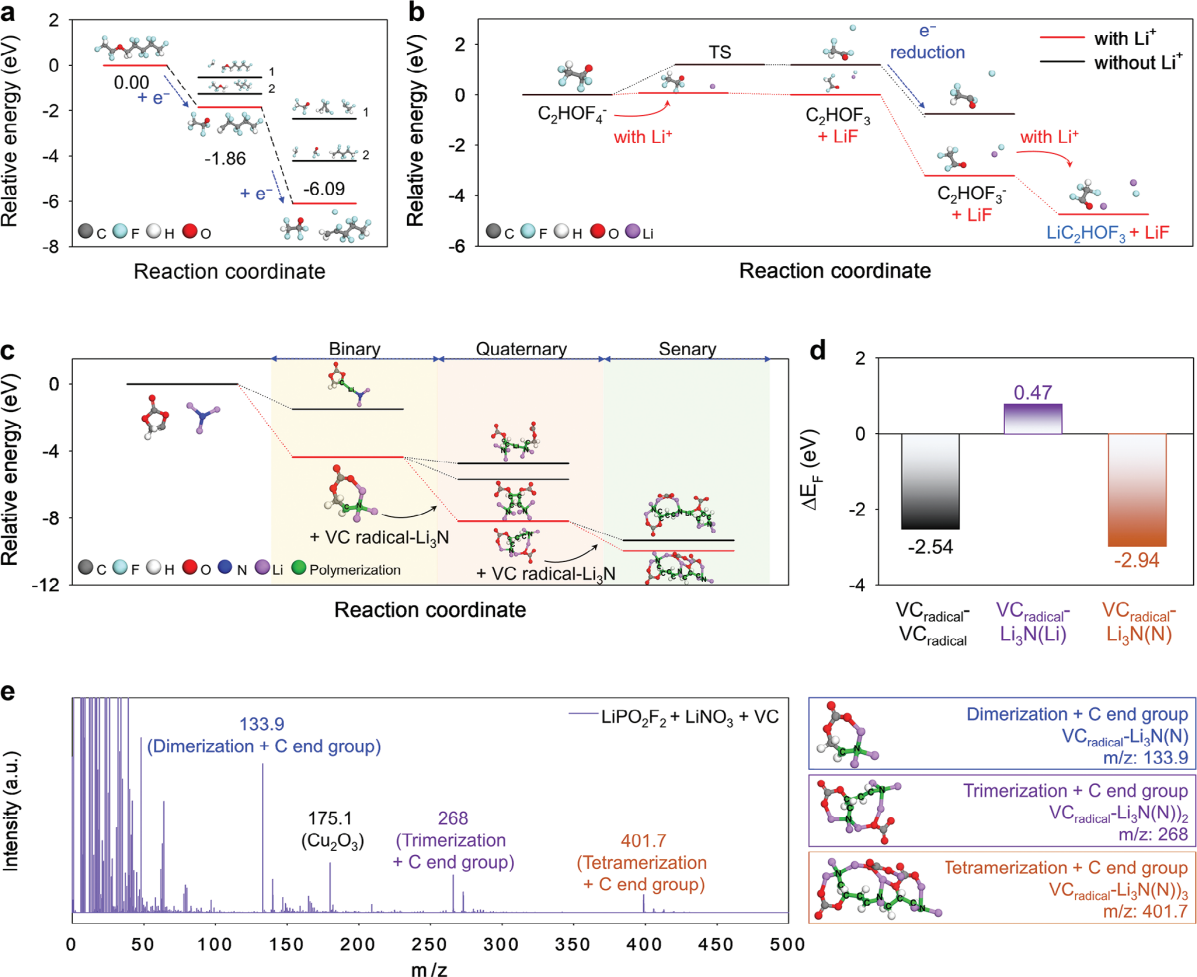
a) Reaction path for TFOFE decomposition by one‐ and two‐electron reduction. b) Reaction path for LiC_2_HOF_3_ and LiF formation by the decomposition of C_2_HOF_4_^−^. c) Relative‐energy diagram of the polymeric reaction with VC radicals and Li_3_N as starting materials. The binary‐ring structure (VC radical–Li_3_N) continuously undergoes reactions to form quaternary and senary structures. d) Formation energy (Δ*E*_F_) values of VC radical–VC radical, VC radical–Li_3_N(Li), and VC radical–Li_3_N(N); the elements written in parenthesis (i.e., Li and N) indicate the element that reacts with the C atom of VC radicals. e) TOF–SIMS pattern of the LiPO_2_F_2_ + LiNO_3_ + VC electrolyte.

TFOFE, which contains F atoms, showed a lower lowest unoccupied molecular orbital (LUMO) energy and higher adsorption energy toward Li‐metal anodes than the other solvents in the electrolyte (Figure S4, Supporting Information). As a result, TFOFE underwent facile reductive decomposition and was preferentially adsorbed on Li‐metal anodes (Figures S5 and S6, Supporting Information). **Figure** **2**a,b indicates the reductive decomposition of TFOFE to generate LiF and several organic species by accepting electrons from a Li‐metal anode.

Density functional theory (DFT) calculations investigated the reaction pathway of TFOFE, which produced LiF and an organic‐species‐based inner SEI (Figure 2a). The first step, comprising the dissociation of the H_2_C–O bond, was found to be thermodynamically favorable via one‐electron reduction. As the LUMO of dissociated TFOFE was energetically lower than that of neutral TFOFE (Figure S7, Supporting Information), a two‐electron reduction process induced C–F bond dissociation in the second step. Further reduction was not expected owing to the higher energy of the LUMO of the two‐electron‐reduced TFOFE species compared to that of neutral TFOFE (Figure S7, Supporting Information). Notably, charge‐analysis calculations indicated that the two electrons injected into the system during reduction were almost evenly distributed on the C_2_HOF_4_ radical and F atom (Figure S8, Supporting Information). Consequently, C_2_HOF_4_^−^ and F^−^ were expected to interact with Li^+^, contributing toward the construction of an organic‐species‐containing SEI layer and LiF, respectively. To confirm this prediction, the decomposition mechanism of C_2_HOF_4_^−^ was investigated (Figure 2b). In the energetics of C_2_HOF_4_^−^ decomposition with and without Li^+^, the diverse adsorption positions of Li^+^ on C_2_HOF_4_^−^ were analyzed to identify the most stable Li^+^–C_2_HOF_4_^−^ complex (Figure S9, Supporting Information). In the presence of Li^+^, the activation energy, and heat of the reaction thermodynamically favored for the formation of LiF and C_2_HOF_3_. C_2_HOF_3_, with a lower‐energy LUMO than TFOFE, C_2_HOF_4_^−^, and LiF (Figure S10, Supporting Information), exhibited a high probability of accepting electrons to form C_2_HOF_3_^−^, which interacted with Li^+^ ions to form the Li^+^–C_2_HOF_3_^−^ complex that subsequently underwent decomposition. The ^19^F NMR spectra of the TFOFE solvent supported the DFT‐derived reduction mechanism of TFOFE. Figure S11a (Supporting Information) compares the integrated ratios of the peak located at −93 ppm (relative to the internal reference (C_6_F_6_)), which corresponds to the F‐functional group adjacent to the O atom (–CF_2_–O–) in TFOFE, before and after storage. After storage with Li‐metal, the intensity of the –CF_2_–O– peak at −93 ppm apparently decreased (Figure S11b, Supporting Information). In addition, a new peak appeared at around −39 ppm after the solvent was stored with the Li‐metal for seven days (Figure S11c, Supporting Information). This result indicates that the TFOFE solvent underwent reductive decomposition by accepting electrons from the Li‐metal, leading to CH_2_–O bond breaking, which resulted in the formation of LiC_2_HOF_3_. The preferential decomposition of TFOFE was also confirmed through a cyclic voltammetry experiment of Li||Cu cells. Electrochemical reductive decomposition of TFOFE was observed at ≈1.7 V versus Li/Li^+^ prior to the reduction of 1,2‐dimethoxyethane (DME) and FSI^–^ (Figure S12, Supporting Information). This preferential reduction caused the defluorination of TFOFE and facilitated the formation of LiF and an organic‐based inner SEI, which effectively inhibited unintended reactions between Li‐metal and solvents such as DME and VC (Figure S13, Supporting Information). Notably, the strong electron‐withdrawing ability of TFOFE, comprising F‐containing functional groups, weakened the interactions in the DME–Li^+^ complex. This increased the proportion of contact ion pairs and cation‐anion aggregate species (AGG‐1 and AGG‐2) in the solvation structure of the electrolyte (Figure S1, Supporting Information), decreasing the participation of DME in the primary sheath of the solvation structure and improving the oxidative endurance of the bulk electrolyte (Figure S14, Supporting Information). Therefore, during charging systems containing Li and LiF, the organic‐species‐based inner SEI layer donated electrons that caused the reductive decomposition of LiPO_2_F_2_, LiNO_3_, and VC. Owing to adsorption‐energy differences, after TFOFE decomposition, the reduction of LiPO_2_F_2_ formed a highly ionically conductive P–O‐based middle SEI layer on the Li‐metal electrode. DFT calculations were used to identify the formation of ionically conductive polymer‐based outer SEI structures comprising repeating units from VC radicals and Li_3_N (Figure 2c). Although the reaction between VC radicals and Li_3_N did not favor C‐Li bond formation (Figure 2d), the VC‐radical C atoms reacted rapidly with the N atoms in Li_3_N molecules, leading to the dissociation of the C‐O bond in VC. Subsequently, these O atoms interacted with the Li atoms in Li_3_N molecules to form binary‐ring structures (VC radical–Li_3_N) that underwent dimerization to form trans‐type quaternary double‐ring structures (Li_3_N–VC radical–VC radical–Li_3_N). Notably, energy calculations (i.e., −2.54 eV) showed comparable formation energies for the VC radical–VC radical and binary‐structure species, indicating quaternary‐structure formation by a thermodynamically stable reaction. Finally, polymeric senary structures were formed by H‐C–C‐H bond formation between the quaternary‐ and binary‐structure species. As H‐C–C‐H bond formation induced the formation of polymeric senary structures, polymeric structures with higher degrees of polymerization were expected to follow a similar trend of reactivity. Moreover, all the aforementioned reactions were spontaneous; thus, the previously mentioned polymeric structures and their conjugates were assumed to contribute toward the construction of the outer layer of the multilayer SEI. In Figure 2e, Time‐of‐flight–secondary ion mass spectrometry (TOF–SIMS) analyzed the formation of repeating units of polymeric species generated by LiPO_2_F_2_ + LiNO_3_ + VC in the electrolyte. Constant signals at m/z = 133.9, 268, and 401.7, corresponding to species formed via the polymerization of LiNO_3_ and VC (through the C end group), were recorded for the polymeric outer SEI layer. DFT calculations and XPS analysis indicated the generation of signals at m/z = 132.5, 268, and 401.7, corresponding to Li_3_C_3_O_3_H_3_N–C, (Li_3_C_3_O_3_H_3_N)_2_–C_2_, and (Li_3_C_3_O_3_H_3_N)_3_–C_3_, respectively, which possibly contributed toward the development of the polymeric outer SEI layer (Figure S15, Supporting Information). *N*otably, the TOF–SIMS depth profiling results elucidated the contribution of the polymer species formed by the polymerized VC radicals and Li_3_N to the outer SEI formation as well as helped to interpret the formation mechanism of the compositionally sequenced SEI layer on the Li‐metal anode (Figure S16, Supporting Information). We could distinctly identify the created polymeric outer SEI, P–O‐based middle SEI, and LiF + organic‐based inner SEI on the Li‐metal anode. The spectra of the additive‐free and LiPO_2_F_2_ + LiNO_3_ electrolytes did not contain any signals above m/z = 100 except for that of copper oxide (Cu_2_O_3_, m/z = 175.1) owing to their inability to form polymeric species on the Li‐metal electrode (Figure S17, Supporting Information). TOF–SIMS was introduced to scrutinize the compositional structure of the polymeric outer SEI layer generated by LiNO_3_ and VC. The multilayer SEI formed in the LiPO_2_F_2_ + LiNO_3_ + VC electrolyte consisted of an inner SEI layer comprising 57.3% of C, 29.9% of F, and 12.8% of O from LiF and several organic species and an outer SEI layer comprising relatively lower atomic percentages of C and F (42.5% and 20.7%, respectively). Moreover, the outer polymeric SEI layer developed in the LiPO_2_F_2_ + LiNO_3_ + VC electrolyte contained higher atomic percentages of O and N than the inner SEI layer (Figure S18, Supporting Information). XPS indicated the construction of an organic‐rich SEI layer comprising R‐Li, C = O, and Li_2_CO_3_ (Figure S19, Supporting Information) in the additive‐free and LiPO_2_F_2_ + LiNO_3_ electrolytes, which contained a lower percentage of LiF than the multilayer SEI generated in the LiPO_2_F_2_ + LiNO_3_ + VC electrolyte (Figure S20, Supporting Information). Notably, the additive‐free and LiPO_2_F_2_ + LiNO_3_ electrolytes led to S‐based SEI formation by the decomposition of FSI^−^ ions and organic species (Figure S21, Supporting Information); the SEI generated in the LiPO_2_F_2_ + LiNO_3_ electrolyte mainly comprised Li_3_N and organic species (Figure S22, Supporting Information).

**Figure 3:**
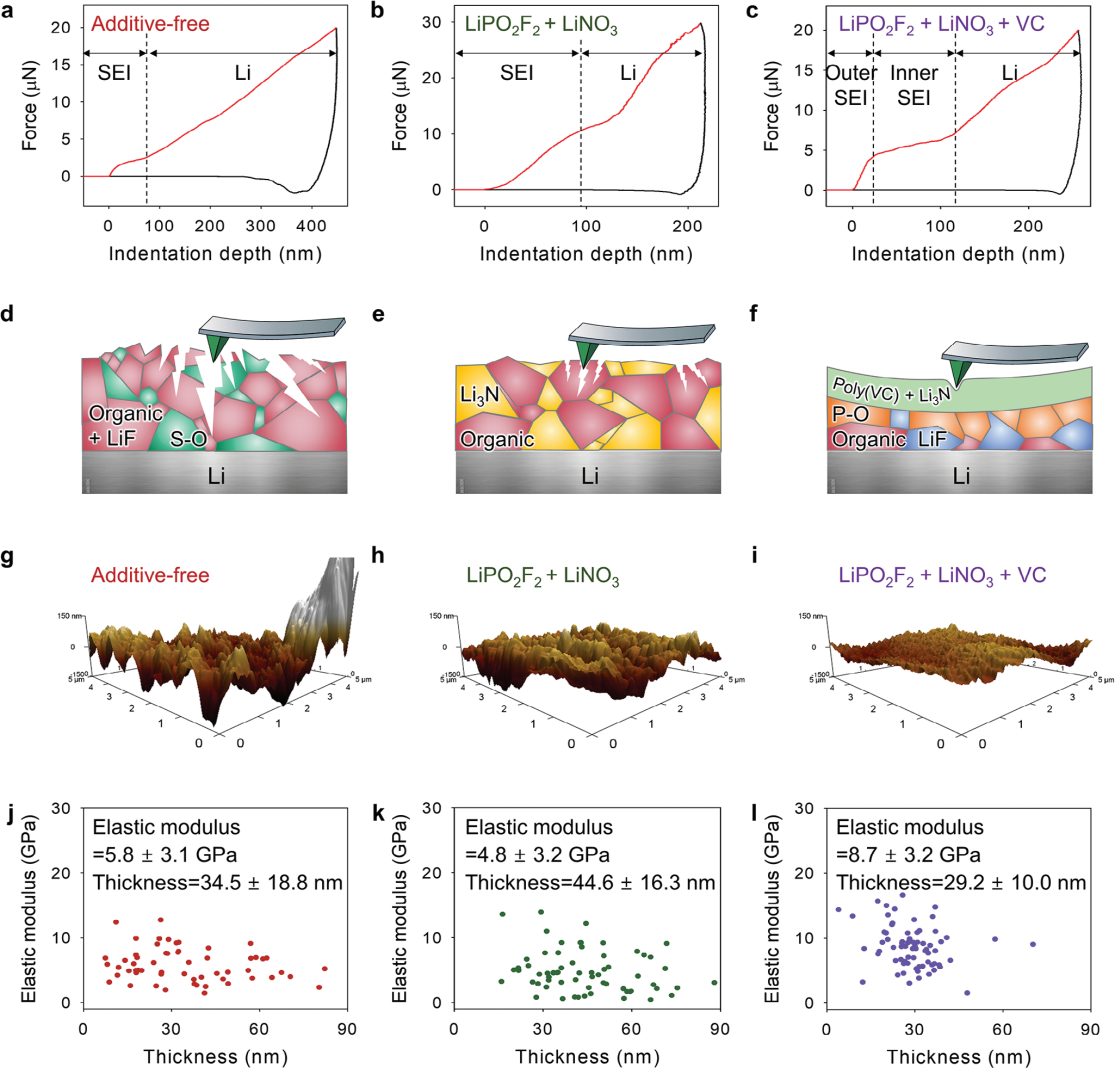
Typical force–displacement curves of Li‐metal anodes in the a) additive‐free, b) LiPO_2_F_2_ + LiNO_3_, and c) LiPO_2_F_2_ + LiNO_3_ + VC electrolytes after precycling. Schematics of the mechanical properties of SEI layers formed in the d) additive‐free, e) LiPO_2_F_2_ + LiNO_3_, and f) LiPO_2_F_2_ + LiNO_3_ + VC electrolytes. AFM 3D images recorded before the measurement of data used for the construction of force–indentation depth curves in the g) additive‐free, h) LiPO_2_F_2_ + LiNO_3_, and i) LiPO_2_F_2_ + LiNO_3_ + VC electrolytes. The elastic moduli and thicknesses of SEI layers formed in the j) additive‐free, k) LiPO_2_F_2_ + LiNO_3_, and those of the polymeric outer SEI layer formed in l) LiPO_2_F_2_ + LiNO_3_ + VC electrolytes.

The mechanical characteristics of the SEI layers formed in different electrolytes were identified by atomic force microscopy (AFM) nanoindentation (**Figure** **3**a–c). The elastic moduli from the SEI layer to the Li–metal were measured by the AFM tip, which touched the Li‐metal after penetrating through the SEI layer. Since the SEI layer and Li‐metal showed different elastic moduli, the mechanical strengths and elastic moduli of the SEI layers formed in different electrolytes differed from each other. The mechanical strength and elastic moduli of the SEI generated in the additive‐free, LiPO_2_F_2_ + LiNO_3_, and LiPO_2_F_2_ + LiNO_3_ + VC electrolytes after precycling were investigated by AFM. The additive‐free and LiPO_2_F_2_ + LiNO_3_ electrolytes generated an amorphous SEI layer that could not tolerate compressive forces, whereas LiPO_2_F_2_ + LiNO_3_ + VC generated a multilayer SEI comprising a polymeric outer SEI, which suppressed spatial deformation and showed high resistivity toward compressive forces, and an inner SEI with a relatively low modulus. Figure 3d–f shows schematics indicating the structural stability of the SEI layers constructed in different electrolytes. Amorphous SEI layers constructed in the additive‐free and LiPO_2_F_2_ + LiNO_3_ electrolytes were unable to adjust to the large volumetric fluctuation of Li‐metal anodes during Li plating because of their mechanical fragility against compressive forces. Contrarily, multilayer SEIs (comprising an inner SEI with a low elastic modulus and a polymeric outer SEI with a high elastic modulus) generated in the LiPO_2_F_2_ + LiNO_3_ + VC electrolyte showed high structural stability on compression. The polymeric outer SEI layer with high elastic modulus dissipated the significant volume stress of Li‐metal anodes. 3D AFM images confirmed that Li‐metal anodes with the multilayer SEI formed in the LiPO_2_F_2_ + LiNO_3_ + VC electrolyte showed a smooth surface after precycling (Figure 3i). Contrarily, Li‐metal anodes containing the excessively deformable and fragile amorphous SEI layer formed in the additive‐free and LiPO_2_F_2_ + LiNO_3_ electrolytes showed rough and uneven surfaces on account of the inability of the SEI layer to endure the localized volume stress induced by Li plating/stripping during precycling (Figure 3g,h). The relation between the elastic modulus and thickness of the different groups of SEI layers shown in Figure 3j–l indicates that the properties and arrangement of the SEI constituents can be tuned to modulate the mechanical robustness of SEI layers; these SEI layers exhibit several advantageous effects such as the dissipation of the volumetric stress induced by Li plating and the inhibition of the vertical growth of Li‐metal. According to AFM‐nanoindentation analysis, the SEI layer constructed in the LiPO_2_F_2_ + VC electrolyte showed a lower elastic modulus (5.6 GPa) than that formed in the LiPO_2_F_2_ + LiNO_3_ + VC electrolyte (8.7 GPa), indicating that the former was mechanically softer than the latter and unable to endure the severe volumetric changes of Li‐metal anodes during cycling (Figure S23a, Supporting Information). AFM 3D images indicated that Li‐metal anodes with LiPO_2_F_2_ + VC‐derived SEIs showed an inhomogeneous and uneven topography (Figure S23b, Supporting Information). Owing to the formation of C–N‐containing polymer‐like structures by the electro‐polymerization of LiNO_3_ and VC in the LiPO_2_F_2_ + LiNO_3_ + VC electrolyte, which inhibits SEI‐layer breakage by the dissipation of volume stress, the SEI layers formed in this electrolyte were mechanically robust. Inconclusive results were obtained by using force‐indentation depth curves for elastic‐modulus fitting and analysis (Figures S24 and S25, Supporting Information). The outliers of nanoindentation over 6 × 6 grid points could not be fit using the Hertzian model, and some graphs showed fluctuating behavior during the approach of the tip, possibly due to electrostatic interaction between the charged spot and tip. Furthermore, local plastic deformation resulted in SEI‐breakage behavior and incomplete force‐indentation depth curves.^[^ ^49^ ^]^ A few force‐indentation depth curves showed a single‐layer behavior, possibly due to the direct exposure of Li‐metal; they were excluded after fitting if the fitted modulus was similar to that of Li‐metal.

### 2.2 Electrochemical Performance of Li‐Metal Anodes with Multilayer SEI

**Figure 4:**
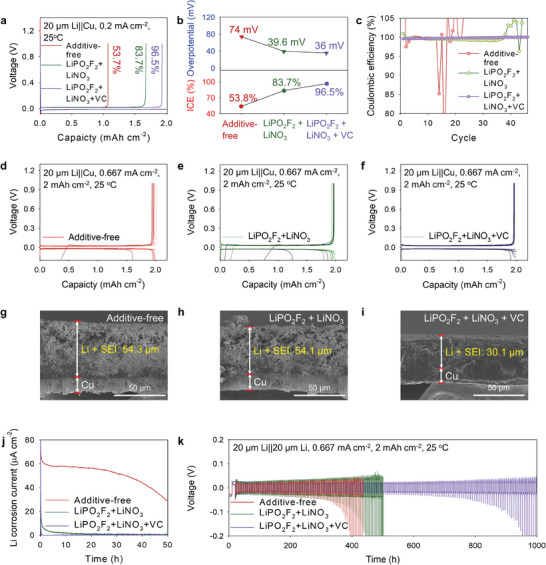
a) Voltage curves of initial Coulombic efficiency in Li||Cu cells. b) ICE and nucleation overpotentials for deposition of Li‐metal in Li||Cu cells. c) CEs of Li||Cu cells including different electrolytes. Voltage curves of Li||Cu cells containing the d) additive‐free, e) LiPO_2_F_2_ + LiNO_3_, and f) LiPO_2_F_2_ + LiNO_3_ + VC electrolytes at 25 ° C. Cross‐sectional scanning electron micrographs of Li‐metal electrodes extracted from Li||Cu cells containing the g) additive‐free, h) LiPO_2_F_2_ + LiNO_3_, and i) LiPO_2_F_2_ + LiNO_3_ + VC electrolytes after the 10^th^ Li‐plating cycle at 0.667 mA cm^−2^ and 2 mAh cm^−2^. j) Galvanic corrosion rates of Li electrodes extracted from Li||Cu cells containing the aforementioned electrolytes. k) Voltage curves of Li plating/stripping in Li||Li cells containing the aforementioned electrolytes at 0.667 mA cm^−2^ and 2 mAh cm^−2^.

**Figure** **4** shows the electrochemical properties of Li||Cu and Li||Li symmetric cells with different electrolytes. A low initial Coulombic efficiency (ICE) of 53.7% with a large overpotential of 74 mV was observed during the Li plating of a Cu current collector in Li||Cu cells containing the additive‐free electrolyte (Figure 4a,b); the addition of LiNO_3_ and VC into the electrolyte improved the ICE to 87.2% and 88.3%, respectively (Figure S26, Supporting Information). However, as the SEI built in the electrolyte respectively including LiNO_3_ and VC was unable to accommodate the compressive force generated by the vertical growth of Li‐metal, it caused low‐density and thick Li plating (Figure S27, Supporting Information). The mechanically fragile SEI formed by electrolytes containing only LiNO_3_ or VC underwent facile breakage, causing a rapid increase in the overpotential of the system after 500 h (Figure S28, Supporting Information). The labile of the SEI layer produced in the additive‐free and LiPO_2_F_2_ + LiNO_3_ electrolytes hindered the reversibility of Li by enabling the continuous corrosion of Li‐metal and facilitating unintended reactions of the electrolytes at the active surface of the Li‐metal electrode (Figure 4c–e,j). Contrarily, the multilayered SEI generated in the LiPO_2_F_2_ + LiNO_3_ + VC electrolyte showed a significantly high ICE of 96.5%, a low overpotential (Figure S29, Supporting Information), good cycling stability, and a high CE value of ≈99.8%, indicating facile Li plating/stripping on Cu substrates (Figure 4c,f). Compared to LiPO_2_F_2_ + LiNO_3_ + VC, LHCE–1,1,2,2,‐tetrafluoroethyl 2,2,3,3‐tetrafluoropropyl ether (TTE) showed a large overpotential of 59.8 mV for the initial Li plating (FigureS30, Supporting Information) and exhibited a relatively low CE of <99.5% during cycling. These results demonstrate that achieving reversible Li plating and stripping using LHCE–TTE is challenging (Figure S31, Supporting Information). Notably, the SEI layer developed in the LiPO_2_F_2_ + LiNO_3_ + VC electrolyte effectively prevented Li corrosion and suppressed Li‐dendrite formation. Thus, cells containing the LiPO_2_F_2_ + LiNO_3_ + VC electrolyte showed low dendritic‐Li formation and dense Li morphology and formed Li and SEI layers with a lower thickness than those formed in the additive‐free and LiPO_2_F_2_ + LiNO_3_ electrolytes (Figure 4g–i; Figure S32, Supporting Information). Notably, the Li electrode in cells including the additive‐free and LiPO_2_F_2_ + LiNO_3_ electrolytes showed an extremely porous Li morphology with thick Li and SEI layers on cycling, indicating that the SEI layers constructed in these systems could not withstand repetitive Li plating/stripping. Uncontrolled Li plating, resulting in the evolution of a less‐compact Li‐plating morphology, was observed in cells containing the additive‐free electrolyte under different areal capacities (Figure S33a, Supporting Information). Notably, partially compressed regions were discovered in the Li‐metal electrode in cells containing the LiPO_2_F_2_ + LiNO_3_ electrolyte, indicating non‐uniform vertical Li growth (Figure S33b, Supporting Information), whereas uniform and compact Li deposition without any compressed regions was observed on the Li‐metal in cells containing the LiPO_2_F_2_ + LiNO_3_ + VC electrolyte under different areal capacities (Figure S33c, Supporting Information). Thus, the hierarchically structured SEI developed in the LiPO_2_F_2_ + LiNO_3_ + VC electrolyte could endure the large volume fluctuation in the Li–metal electrode caused by increments in the areal capacity, imparting Li||Li symmetric cells with an excellent cycling stability (Figure 4k). Notably, Li||Li symmetric cells with the LiPO_2_F_2_ + LiNO_3_ + VC electrolyte showed a low overpotential of < 100 mV for 1000 h, corresponding to 180 cycles, indicating the mitigation of electrode–electrolyte side reactions during Li plating/stripping.

### 2.3 Electrochemical Performance of LMBs with Developed Electrolytes

**Figure 5:**
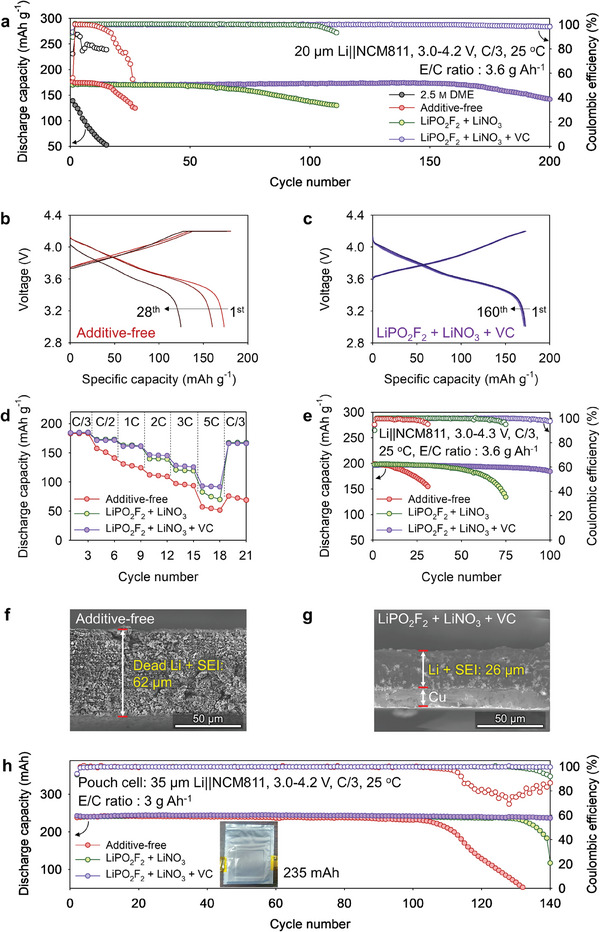
a) Cyclic stability of Li||NCM811 full cells with 2032‐coin‐type. Voltage curves of Li||NCM811 full cells containing the b) additive‐free and c) LiPO_2_F_2_ + LiNO_3_ + VC electrolytes during cycling. d) Rate performances of the aforementioned cells with diverse charge rates and constant discharge rate of C/3. e) Cyclic stability of Li||NCM811 full cells with 2032‐coin‐type. Cross‐sectional scanning electron micrographs of Li‐metal anodes extracted from Li||NCM811 full cells containing the f) additive‐free and g) LiPO_2_F_2_ + LiNO_3_ + VC electrolytes after 50 cycles. h) Cyclic stability of Li||NCM811 pouch cells at 235 mAh. Inset: Digital photograph of the as‐fabricated pouch cell.

The oxidation durability of different electrolytes was compared by Linear sweep voltammetry (LSV) in Li||Al cells and electrochemical floating tests in Li||NCM811 full cells at 4.2 V versus Li/Li^+^. The LiPO_2_F_2_ + LiNO_3_ and LiPO_2_F_2_ + LiNO_3_ + VC electrolytes showed high onset‐voltage values, indicating better oxidation stability than that of the additive‐free electrolyte (Figure S34, Supporting Information). Leakage‐current measurements indicated that the LiPO_2_F_2_ + LiNO_3_ + VC electrolyte constructed a protective CEI on the NCM811 cathode that minimized parasitic unintended reactions between the cathode and electrolyte (Figure S35, Supporting). To investigate the compatibility of the additive‐free, LiPO_2_F_2_ + LiNO_3_, and LiPO_2_F_2_ + LiNO_3_ + VC electrolytes with Li‐metal and NCM811 cathodes, 20 µm Li||NCM811 full cells with each of the aforementioned electrolytes were subjected to electrochemical cycling under the lean‐electrolyte system (E/C ratio: 3.6 g Ah^−1^) (Table S2, Supporting Information). Notably, in **Figure** **5**a, the full cells containing the LiPO_2_F_2_ + LiNO_3_ + VC electrolyte showed excellent cycling stability with a superior average CE of 99.9% over 160 cycles. The optimal contents of VC and LiNO_3_ in the LiPO_2_F_2_ + LiNO_3_ + VC electrolyte were determined to be 1 wt.% each from the cycling results of the full cells with different contents of VC (within 0–2 wt.%) (Figure S36, Supporting Information) and LiNO_3_ (within 0–1.5 wt.%) (FigureS37, Supporting Information), respectively. With cycling, the constant‐voltage (CV) charging period of cells containing the additive‐free and LiPO_2_F_2_ + LiNO_3_ electrolytes became longer and their discharge capacities rapidly deteriorated (Figure 5b; Figure S38, Supporting Information), indicating that the electrochemical stability of these electrolytes toward NCM811 cathodes and Li‐metal was insufficient for prolonged CV charging and could not inhibit parasitic electrode‐electrolyte side reactions. The accumulation of byproducts owing to continual electrolyte degradation caused an increase in the overpotential of the full cells, lengthening the CV mode and shortening the constant‐current period. Contrarily, the LiPO_2_F_2_ + LiNO_3_ + VC electrolyte effectively limited the CV‐charging‐mode duration in Li||NCM811 full cells, enabling them to exhibit an excellent cycling performance (over 160 cycles) without significant capacity degradation (Figure 5c). Interfacial‐resistance changes were used to investigate the stability of interfacial layers formed in the LiPO_2_F_2_ + LiNO_3_ + VC electrolyte (Figure S39, Supporting Information). The interfacial resistance of full cells containing the additive‐free electrolyte, which underwent uncontrolled electrolyte decomposition at the electrodes, could not be measured after 100 cycles, possibly because the high electrolyte resistance caused by severe electrolyte depletion in the full cell made electrolyte‐resistance measurements challenging, even at high frequencies. Full cells containing the LiPO_2_F_2_ + LiNO_3_ + VC electrolyte showed a negligible interfacial‐resistance increment compared to those containing the LiPO_2_F_2_ + LiNO_3_ electrolyte. Notably, the low interfacial resistance of the LiPO_2_F_2_ + LiNO_3_ + VC electrolyte enabled the Li||NCM811 full cells to exhibit an improved fast charging performance and a discharge rate of C/3 (Figure 5d). Therefore, because of the low interfacial resistance and high ionic conductivity of the LiPO_2_F_2_ + LiNO_3_ + VC electrolyte, the rate performance of the Li||NCM811 full cells containing this electrolyte surpassed that of the cells containing LHCE–TTE, which exhibited a low ionic conductivity; consequently, this superior electrolyte weakened the capacity retention of the Li||NCM811 full cells at high C‐rate conditions (Table S1; Figures S39 and S40, Supporting Information). Additionally, through the interfacial stability of the electrodes, the LiPO_2_F_2_ + LiNO_3_ + VC electrolyte enabled the full cells to exhibit good cycling stability at a high charge‐voltage cut‐off of 4.3 V versus Li/Li^+^ (Figure 5e). LiPO_2_F_2_ underwent oxidative decomposition to form P–O species (as CEI constituents) on the NCM811 cathode (Figure S41, Supporting Information), thereby improving the interfacial stability of the NCM811 cathode and suppressing the oxidative degradation of the electrolyte under high‐voltage conditions. CEIs containing polar P–O species exhibit facile Li^+^‐ion migration. Therefore, full cells containing the LiPO_2_F_2_ + LiNO_3_ + VC electrolyte showed a good cycling performance at 1C (Figure S42, Supporting Information). The multilayer SEI developed in the LiPO_2_F_2_ + LiNO_3_ + VC electrolyte maintained a dense Li‐metal anode of 26 µm after 50 cycles; contrarily, cells containing the additive‐free electrolyte contained a thick and porous Li‐metal anode with dead Li and the byproducts of electrolyte decomposition (Figure 5f,g). Similar to the electrodes in cells containing the additive‐free electrolyte, those in cells containing the LiPO_2_F_2_ + LiNO_3_ electrolyte showed a less compact and inhomogeneous Li morphology (Figure S43, Supporting Information). Notably, Li||NCM811 pouch cells containing the LiPO_2_F_2_ + LiNO_3_ + VC electrolyte designed in the lean‐electrolyte system of 3 g Ah^−1^ showed better cyclic stability than those containing the additive‐free and LiPO_2_F_2_ + LiNO_3_ electrolytes, which showed rapid capacity fading (Figure 5h).

### 2.4 Effect of LiPO_2_F_2_‐Driven CEI on Microstructures of NCM811 Cathodes

**Figure 6:**
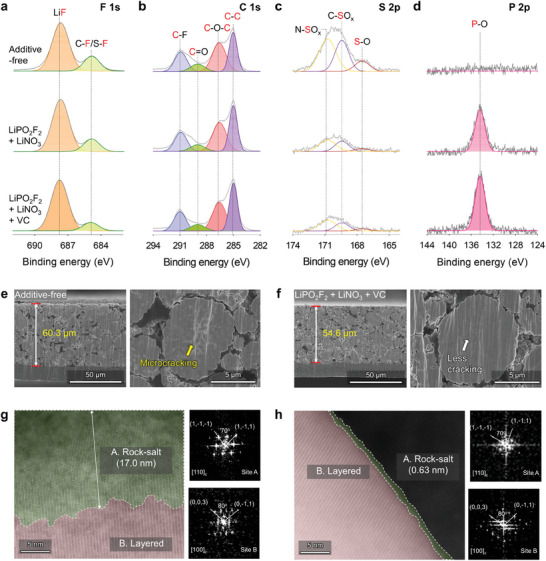
a) F 1s, b) C 1s, c) S 2p, and d) P 2p XPS spectra of NCM811 cathodes precycled in different electrolytes. Cross‐sectional SEM images of NCM811 cathodes extracted from Li||NCM811 full cells containing the e) additive‐free and f) LiPO_2_F_2_ + LiNO_3_ + VC electrolytes after 200 cycles at C/3 and 25 ° C. STEM images with FFT patterns of NCM811 cathodes extracted from full cells containing the g) additive‐free and h) LiPO_2_F_2_ + LiNO_3_ + VC electrolytes after 200 cycles at C/3 and 25 ° C.

The LiPO_2_F_2_ + LiNO_3_ + VC electrolyte not only improved the SEI quality on the Li‐metal anodes in Li||NCM811 full cells but also modified the CEI constitution on the NCM811 cathodes in the system (**Figure** **6**). P 2p spectra indicated that the additive‐free electrolyte did not generate P–O species on the cathode, while the LiPO_2_F_2_ + LiNO_3_ and LiPO_2_F_2_ + LiNO_3_ + VC electrolytes generated a P–O‐based CEI layer that facilitated rapid Li^+^‐ion transfer and minimized parasitic reactions between the NCM811 cathode and electrolyte. Notably, the LiPO_2_F_2_ + LiNO_3_ and LiPO_2_F_2_ + LiNO_3_ + VC electrolytes effectively alleviated the construction of *S*, organic‐containing species by the oxidative degradation of the LiFSI salt and DME solvent at the cathode (Figure 6a–d). The C 1s XPS spectrum of the LiPO_2_F_2_ + LiNO_3_ + VC electrolyte after precycling contained a high‐intensity C–C peak at 285 eV, indicating the development of a thin CEI layer that preserved the electrical conducting network of the binder and the electrical conducting agent. Inductively coupled plasma–optical emission spectrometry (ICP–OES) identified the ability of the CEI formed in the LiPO_2_F_2_ + LiNO_3_ + VC electrolyte to enhance the structural durability of NCM811 cathodes. The LiPO_2_F_2_ + LiNO_3_ + VC electrolyte mitigated the dissolution of Ni, Co, Mn, and Al from delithiated NCM811 cathodes stored at 60 ° C for 3 days to a greater extent than the other electrolytes (Table S3 and Figure S44, Supporting Information). The morphological stabilization of NCM811 cathodes cycled in the LiPO_2_F_2_ + LiNO_3_ + VC electrolyte was examined by cross‐sectional SEM analysis (Figure 6e,f). Intragranular cracking was not observed in the secondary particles of these NCM811 cathodes, indicating that the LiPO_2_F_2_‐induced CEI in the system enabled uniform Li extraction and NCM secondary‐particle insertion, dissipating the inhomogeneous structural stress that causes microcracking. Contrarily, the additive‐free electrolyte led to inhomogeneous CEI formation on the cathode, resulting in different levels of lithiation/delithiation in the NCM primary particles, thereby aggravating the mechanical disintegration of the secondary particles. X‐ray diffraction (XRD) confirmed the low structural stability of NCM811 cathodes cycled in the additive‐free electrolyte (Figure S45, Supporting Information). On cycling NCM811 cathodes in the additive‐free electrolyte, the (003) peak in the XRD pattern of pristine NCM811 cathodes shifted to the left, while the (110) and (113) peaks shifted to the right; significantly lesser peak shifts were detected on cycling the cathode in the LiPO_2_F_2_ + LiNO_3_ + VC electrolyte. Thus, the LiPO_2_F_2_ + LiNO_3_ + VC electrolyte prevented the structural deterioration of NCM811 cathodes through the formation of a highly stable CEI layer. Scanning transmission electron microscopy (STEM) with fast Fourier transform (FFT) confirmed the ability of the LiPO_2_F_2_ + LiNO_3_ + VC electrolyte to mitigate the irreversible phase transition of NCM811 cathodes from the layered to the rock‐salt structure (Figure 6g,h). Contrarily, NCM811 cathodes underwent significant structural degradation in the additive‐free electrolyte, forming a 17 nm thick rock‐salt phase. This irreversible phase transition to the rock‐salt phase adversely affected Li^+^‐ion transport in the bulk‐cathode structure, significantly reducing the Li^+^‐storage capability of the system. The rock‐salt phase was possibly generated by the reduction of the highly reactive Ni^4+^ species to Ni^3+^ and Ni^2+^ in the delithiated state; the resulting Ni^2+^, with a similar size as Li^+^, migrated into the Li slab, resulting in Li/Ni cation mixing. This phenomenon was not observed in the LiPO_2_F_2_ + LiNO_3_ + VC electrolyte, which caused very little rock‐salt phase formation owing to the suppression of the aforementioned irreversible phase transition.

## 3 Conclusion

This study reports the design and development of a compositionally sequenced multilayer SEI comprising LiF, P–O‐containing molecules, and polymeric species and a LiPO_2_F_2_‐driven CEI that enables the stable cycling of Li||NCM811 full cells under lean‐electrolyte conditions. The electrically insulating polymeric outer layer of the newly proposed multilayer SEI reduced the probability of the deposition of Li‐metal on the upper surface of the SEI, enhancing the electrochemical reversibility of the Li‐metal anode. Moreover, the electrochemically and mechanically robust inner layer of the multilayer SEI enabled dense Li plating without porous‐structure‐induced cell swelling and minimal lateral Li plating on the anode. The newly proposed LiPO_2_F_2_‐induced CEI mitigated the irreversible structural change of the cathode from the layer structure to the inactive rock‐salt structure. This study could lead to the development of electrolyte formulations that minimize the structural deterioration of interfacial layers in LMBs. Additionally, the results in this study enable a comprehensive insight into the composition and structure of the SEI and CEI in LMBs, contributing significantly toward research on the chemistry of battery electrolytes.

## 4 Experimental Section

### DFT Calculations

The DMol^3^ program was used for DFT calculations to evaluate the highest occupied molecular orbital (HOMO) and LUMO, formation energies, adsorption energies, bond‐dissociation energies, and reaction mechanisms.^[^ ^50^, ^51^ ^]^ Depending on the targeted accuracy and effectiveness, the exchange‐correlation energies were calculated using Beck's three‐parameter hybrid function combined with the Lee–Yang–Parr correlation functional or the generalized gradient approximation with the Perdew–Burke–Ernzerhof functional.^[^ ^52^, ^53^, ^54^ ^]^ The effective core potential was used for core treatment with the double numerical plus polarization 4.4 level basis set. The Tkatchenko–Scheffler van der Waals correction method was used for spin‐polarized calculations.^[^ ^55^ ^]^ A convergence criterion of 1 × 10^−6^ Ha was used for self‐consistent calculations, while 1 × 10^−5^ Ha, 0.002 Ha Å^−1^, and 0.005 Å were set as the geometry‐optimization convergence criteria for the maximum energy change, maximum force, and maximum displacement, respectively. The conductor‐like screening model using the dielectric constant of DME (i.e., 7.2) was used to implicitly impose the organic solvent environment used during experimentation.^[^ ^56^, ^57^ ^]^ Complete single linear synchronous transit and quadratic synchronous transit with a root mean square convergence force of 0.002 Ha Å^−1^ on the atoms were used for transition‐state calculations.^[^ ^58^, ^59^ ^]^

The VC radical–VC radical and VC radical–Li_3_N formation energies were calculated by the following equations:

(1) $$\Delta E_{F}=E_{\mathrm{total}}-2E_{\mathrm{VC}}-\frac{1}{2}n\mu_{H_{2}}$$

(2) $$\Delta E_{F}=E_{\mathrm{total}}-E_{\mathrm{VC}}-E_{\mathrm{LiN}O_{3}}-\frac{1}{2}n\mu_{H_{2}}-n\mu_{\mathrm{Li}}+\frac{1}{2}n\mu_{O_{2}}$$

where *E*_total_ represents the total energy of the product, *E*_VC_ represents the total energy of VC, $E_{\mathrm{LiN}O_{3}}$ represents the total energy of LiNO_3_, and $\mu_{H_{2}}$, $\mu_{O_{2}}$, and µ_Li_ indicate the chemical potentials of hydrogen gas, oxygen gas, and Li–metal, respectively, as the reference states. The adsorption energy of a complex formed by two chemicals labeled *i* and *j* was calculated by the following equation:^[^ ^60^ ^]^

(3) $$\Delta E_{\mathrm{Adsorption}}=E_{\mathrm{total}}-\left(E_{i}+E_{j}\right)$$

where *E*_total_ represents the total energy of the two chemicals bound together, *E*_i_ represents the total energy of the Li‐(001)‐surface‐slab model or Li^+^ ions, and *E*_j_ represents the energy of TFOFE, VC, DME, LiFSI, LiNO_3_, LiPO_2_F_2_, or C_2_HOF_4_^−^. The dissociation energy of an *F* atom from C_2_HOF_4_^−^ was calculated by the following equation:

(4) $$\Delta E_{F\;\mathrm{dissociation}}=E_{C_{2}{\mathrm{HOF}}_{4}^{-}}-\left(E_{F}+E_{C_{2}{\mathrm{HOF}}_{3}^{-}}\right)$$

where $E_{C_{2}{\mathrm{HOF}}_{4}^{-}}$, *E*_F_, and $E_{C_{2}{\mathrm{HOF}}_{3}^{-}}$ indicate the total energies of C_2_HOF_4_^−^, an F atom, and C_2_HOF_3_^−^, respectively.

### Electrolyte and Electrode Fabrication

First, the additive‐free electrolyte was prepared by adding LiFSI salt (2.5 M) in DME and TFOFE (8/2 vol%). Subsequently, the other electrolytes were synthesized by adding 0.3 wt.% of LiPO_2_F_2_, 1 wt.% of LiNO_3_, and 1 wt.% of VC into the electrolyte. The LiPO_2_F_2_ + LiNO_3_ electrolyte was prepared by adding 0.3 wt.% of LiPO_2_F_2_ and 1 wt.% of LiNO_3_ into the additive‐free electrolyte, while the LiPO_2_F_2_ + LiNO_3_ + VC electrolyte was prepared by dissolving 0.3 wt.% of LiPO_2_F_2_, 1 wt.% of LiNO_3_, and 1 wt.% of VC in the additive‐free electrolyte. For moisture removal, CaH_2_ was brought into the electrolytes and stirred for 30 min; the Ca(OH)_2_, which was generated by the reaction of CaH_2_ with moisture, was filtered out. To fabricate NCM811 cathodes, a slurry containing 2 wt.% of poly(vinylidene fluoride), 2 wt.% of carbon black, and 96 wt.% of the active material was applied onto Al foils, which were compressed by doctor blade and dehydrated under 110 ° C for 10 h in vacuum oven (thickness: 56 µm; loading level: 17.5 mg cm^−2^; areal capacity: 3.2 mAh cm^−2^).

### Electrochemical Measurements

Coin cells (2032‐coin‐type) with 20 µm Li‐metal anodes (Honjo), ceramic‐coated separators (porosity: 43.7%, thickness: 12 µm), and NCM811 cathodes (loading level: 17.5 mg cm^−2^) were fabricated in an Ar‐filled glove box (H_2_O and O_2_ < 1.0 ppm) and examined for electrochemical measurements. To achieve a high energy density, an electrolyte (20 µL; 3.6 g Ah^−1^) with a low E/C ratio was put into the Li||NCM811 full cells, which were cycled from 3.0 to 4.2 V and 4.3 V versus Li/Li^+^ at 25 °C (WBCS3000, WonATech). The cells were cycled first twice with C/10 for the construction of interfacial layers on the electrodes. After that, the full cells were cycled at C/3. After the 1^st^ and 100^th^ cycles, an electrochemical potentiostat (VSP‐2e, Biologics) was used to measure the cell impedance of the cells over a frequency range from 0.01 to 1 MHz by AC impedance analysis. The full cells in fully charged states, which were sustained at 4.2 V for 10 h, were used for leakage‐current measurements. For rate performance testing, the full cells were discharged at C/3 while the charging rate was increased from C/3 to C/2, 1, 2, 3, and 5C. In the last three cycles, the full cells were cycled with C/3 to evaluate the recovery performance of the device. The electrochemical characteristics of the multilayer SEI formed in different electrolytes were investigated using Li||Li symmetrical cells and Li||Cu cells with 2016‐coin‐type. A Li||Cu cell was prepared to measure the ICE during Li plating and stripping. To enable the development of an SEI layer on the Li‐metal electrode, Li||Cu cells were precycled with 0.2 mA cm^−2^. Subsequently, to examine the reductive durability of the electrolytes, Li‐metal was deposited on a Cu current collector in the cell, which was maintained at 0 V versus Li/Li^+^ for 50 h. The cycling performances of Li||Cu and Li||Li cells were analyzed by precycling and cycling both cells with 0.667 and 2 mAh cm^−2^, respectively.

### Characterization

A glove box was used for all analyses. During analysis, the full cells were dissembled and the electrodes were cleaned with DME (to rinse any adhering electrolyte). A TOF–SIMS5 (ION‐TOF GmbH) was used; a Bi_1_^+^ (25 keV, 1.3 pA) beam was used to analyze a measurement area of 500 × 500 µm. The thickness and elastic modulus values of the SEI layers constructed in the additive‐free, LiPO_2_F_2_ + LiNO_3_, and LiPO_2_F_2_ + LiNO_3_ + VC electrolytes were measured using a commercially available AFM instrument (Oxford Instruments Asylum Research, MFP 3D Origin)^[^ ^61^ ^]^ with a cone‐shaped AFM tip (NC‐LC, Adama Innovations, spring constant: 100 N m^−1^, tip radius: 20 nm) and ORCA holder (dual gain, Oxford Instruments Asylum Research). Topography images of the SEI layers were acquired in the AC mode at frequencies in the range of 450–540 Hz (tuned at +5% from the peak) with a setpoint within 590–740 mV.^[^ ^62^ ^]^ Force–distance curves (rate: 0.1 Hz, range: 1–1.5 µm) were evaluated using 6 × 6 grid points, and the Hertzian model (Asylum Research Software) was used to estimate the elastic moduli of the as‐fabricated SEI layers; the initial 10% of each displacement curve was fitted to minimize the effect of the underlying Li‐metal. All experiments were carried out under Ar (99.9999%) at 25 °C within closed cells. A connected syringe and gas tube were used to control the pressure inside each closed cell. XPS (K‐alpha, Thermo VG Scientific) with Al‐Kα (hv = 1486 eV) X‐rays with ultrahigh vacuum were used to investigate the constituents of the as‐fabricated multilayer SEI and CEI layers. SEM (SU8230, Hitachi) was utilized to identify the cross‐sectional morphologies of the Li‐metal anode and NCM811 cathode in cells containing the additive‐free, LiPO_2_F_2_ + LiNO_3_, and LiPO_2_F_2_ + LiNO_3_ + VC electrolytes. STEM (Titan G2 60–300, FEI) was used to compare the structures of NCM811 cathodes cycled in the additive‐free, LiPO_2_F_2_ + LiNO_3_, and LiPO_2_F_2_ + LiNO_3_ + VC electrolytes. For the STEM, NCM811 cathodes were prepared by a focused ion‐beam system (Helios Nanolab 450, FEI); the cycled NCM811 cathodes were coated with a carbon layer.

## Conflict of Interest

The authors declare no conflict of interest.

## Author Contributions

J.‐A.L., S.K., Y.C., and S.H.K contributed equally to this work. E.K., S.S., W.K., K.H.R., and N.‐S.C. proposed and designed the projects. J.‐A.L., and S.K. conducted characterizations and electrochemical measurements. Y.C., and S.H. analyzed the nanoindentation of SEI layers formed on Li‐metal electrodes. S.H.K., S.K.K. carried out DFT calculations. J.‐A.L., S.K., Y.C., S.H.K., H.K., J.H.B., S.K.K., S.H., and N.‐S.C. wrote the manuscript. All authors analyzed the data and contributed to the discussion.

## Supporting information

Supporting Information

## Data Availability

The data that support the findings of this study are available from the corresponding author upon reasonable request.
