# Supplementary material for: Compositionally Sequenced Interfacial Layers for High‐Energy Li‐Metal Batteries
Source: Adv Sci (Weinh). 2024 Feb 26;11(17):2310094. doi: 10.1002/advs.202310094 (PMC11077642; doi:10.1002/advs.202310094)
Supplement: Supplementary file 1 — Supporting Information [file ADVS-11-2310094-s001.pdf]

ADVANCED SCIENCE
Open Access

Supporting Information

for *Adv. Sci.*, DOI 10.1002/advs.202310094

Compositionally Sequenced Interfacial Layers for High-Energy Li-Metal Batteries

Jeong-A Lee, Saehun Kim, Yoonhan Cho, Seong Hyeon Kweon, Haneul Kang, Jeong Hwan Byun, Eunji Kwon, Samuel Seo, Wonkeun Kim, Kyoung Han Ryu, Sang Kyu Kwak, Seungbum Hong* and Nam-Soon Choi**

Supporting Information

Compositionally Sequenced Interfacial Layers for High-Energy Li-Metal Batteries

Jeong-A Lee, Saehun Kim, Yoonhan Cho, Seong Hyeon Kweon, Haneul Kang, Jeong Hwan Byun, Eunji Kwon, Samuel Seo, Wonkeun Kim, Kyoung Han Ryu, Sang Kyu Kwak,* Seungbum Hong,* and Nam-Soon Choi*

J.-A. Lee, S. Kim, H. Kang, J. H. Byun, Prof. N.-S. Choi

Department of Chemical and Biomolecular Engineering

Korea Advanced Institute of Science and Technology (KAIST)

291 Daehak-ro, Yuseong-gu, Daejeon 34141, Republic of Korea

E-mail: nschoi@kaist.ac.kr

Y. Cho, Prof. S. Hong

Department of Materials Science and Engineering

Korea Advanced Institute of Science and Technology (KAIST)

291 Daehak-ro, Yuseong-gu, Daejeon 34141, Republic of Korea

E-mail: seungbum@kaist.ac.kr

S. H. Kweon

School of Energy and Chemical Engineering

Ulsan National Institute of Science and Technology (UNIST)

50 UNIST-gil, Ulsan 44919, Republic of Korea

E. Kwon, S. Seo, W. Kim, K. H. Ryu

CTO Advanced Battery Development

Hyundai motor company

37 Cheoldobangmulgwan-ro, Uiwang-si, Gyeonggi-do 16082, Republic of Korea

Prof. S. K. Kwak

Department of Chemical and Biological Engineering

Korea University

145 Anam-ro, Seongbuk-gu, Seoul 02841, Republic of Korea

E-mail: skkwak@korea.ac.kr

Experimental Section

Characterization

Inductively coupled plasma–optical emission Spectroscopy (ICP–OES, 700-ES, Varian) was used to investigate the role of the CEI layer formed on the NCM811 cathode in different electrolytes on transition-metal (Ni, Mn, and Co) and Al dissolution from the electrode. The fully charged NCM811 cathodes were disassembled, washed with the solvent DME to remove any residual electrolyte, and stored in an oven at 60 ℃ for 3 days. The structures of the NCM811 cathodes cycled in the additive-free, $LiPO_2F_2$ + $LiNO_3$, and $LiPO_2F_2$ + $LiNO_3$ + VC electrolytes were analyzed by high-resolution thin-film XRD (SmartLab, RIGAKU) using theta(θ)/2theta analysis within 10–80° at a scan rate of 1° min^{-1}. In this study, 2016-coin-type 700-μm Li||Al cells were used for linear sweep voltammetry (LSV) at a scan rate of 1 mV s^{-1} at 25 ℃.

Computational details

The $DMol^3$ software was used for all DFT calculations to investigate the HOMO and LUMO energy levels, formation energies, bond-dissociation energies, and reaction mechanisms. Beck's three-parameter hybrid functional combined with the Lee–Yang–Parr correlation

functional was used for electron exchange-correlation energy calculations. The effective core potential was used for core treatment with the double numerical plus polarization 4.4 level basis set. The Tkatchenko–Scheffler van der Waals correction method was used for spin-polarized calculations. The convergence criterion for self-consistent calculations was set as 1×10^{-6} Ha, and the convergence criteria for geometry optimization were set as 1×10^{-5} Ha, 0.002 Ha $Å^{-1}$, and 0.005 Å for the maximum energy change, maximum force, and maximum displacement, respectively. The organic-solvent environment for analysis was implicitly constructed by the conductor-like screening model using the dielectric constant of 1,2-dimethoxy ethane (DME) (7.2). The reaction-mechanism transition states were calculated using the generalized gradient approximation with the Perdew–Burke–Ernzerhof exchange-correlation functional. The complete single linear synchronous transit and quadratic synchronous transit methods were used for calculations considering 0.002 Ha $Å^{-1}$ to be the root mean square convergence force on the atoms.

Table S1. Ionic conductivities and densities of the electrolytes

	2.5 M LiFSI DME	Additive-free	$LiPO_2F_2$ + $LiNO_3$ + VC	LHCE-TTE	LHCE-TFOFE
Ionic conductivity (mS cm^{-1})	8.26	7.45	7.55	3.65	0.97
Density (g ml^{-1})	1.16	1.29	1.27	1.47	1.58

Table S2. Amount of electrolyte injected into full cells comprising Li-metal anodes and NCM811 cathodes

Amount of electrolyte (15 μl) in Li‖NCM811 full cells	
Per coin cell	0.0177 g $cell^{-1}$
Per capacity of cathode (E/C ratio)	3.6 g Ah^{-1}

Table S3. Results of the ICP–OES analysis of Ni, Co, Mn, and Al dissolution from fully charged (delithiated) NCM811 cathodes in different electrolytes after 3 days of storage at 60 ℃

	Additive-free	$LiPO_2F_2$ + $LiNO_3$	$LiPO_2F_2$ + $LiNO_3$ + VC
Ni (ppm)	2.66	0.95	0.58
Co (ppm)	0.105	0.098	0.041
Mn (ppm)	0.02	0.01	0.01
Al (ppm)	1.17	0.94	0.52

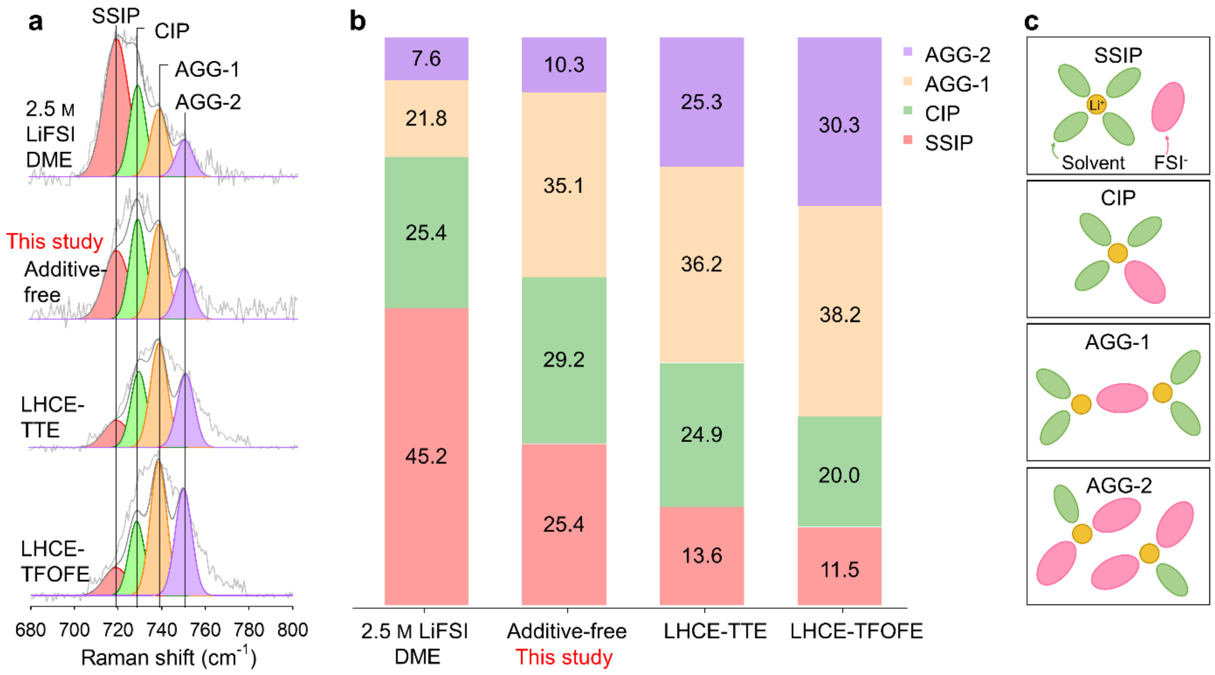

Figure S1. a) Raman spectra of the different electrolytes and b) proportion of solvation separated ion pairs (SSIP), contact ion pairs (CIP), and aggregate (AGG-1 and AGG-2) species of 2.5 M LiFSI DME, additive-free, LHCE–TTE, and LHCE–TFOFE electrolytes. c) Schematic illustration of the species present in the solvation structure. Additive-free: 2.5 M LiFSI DME/TFOFE (8/2 vol%) (base electrolyte used in this study), LHCE-TTE: 1 M LiFSI DME/TTE (2/8 vol%), and LHCE–TFOFE: 1 M LiFSI DME/TFOFE (2/8 vol%).

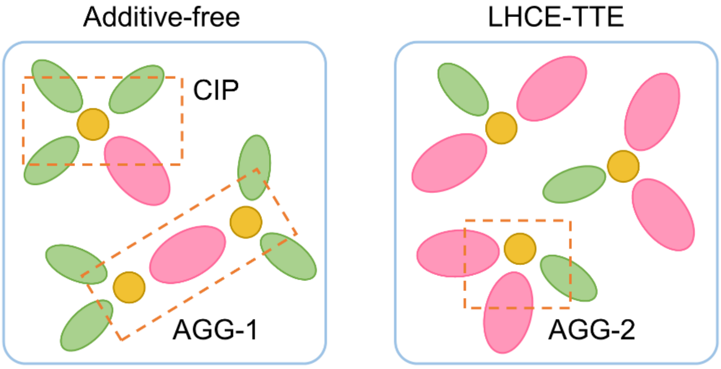

Figure S2. Schematic illustration of the solvation structures of the additive-free (our system) and LHCE–TTE electrolytes.

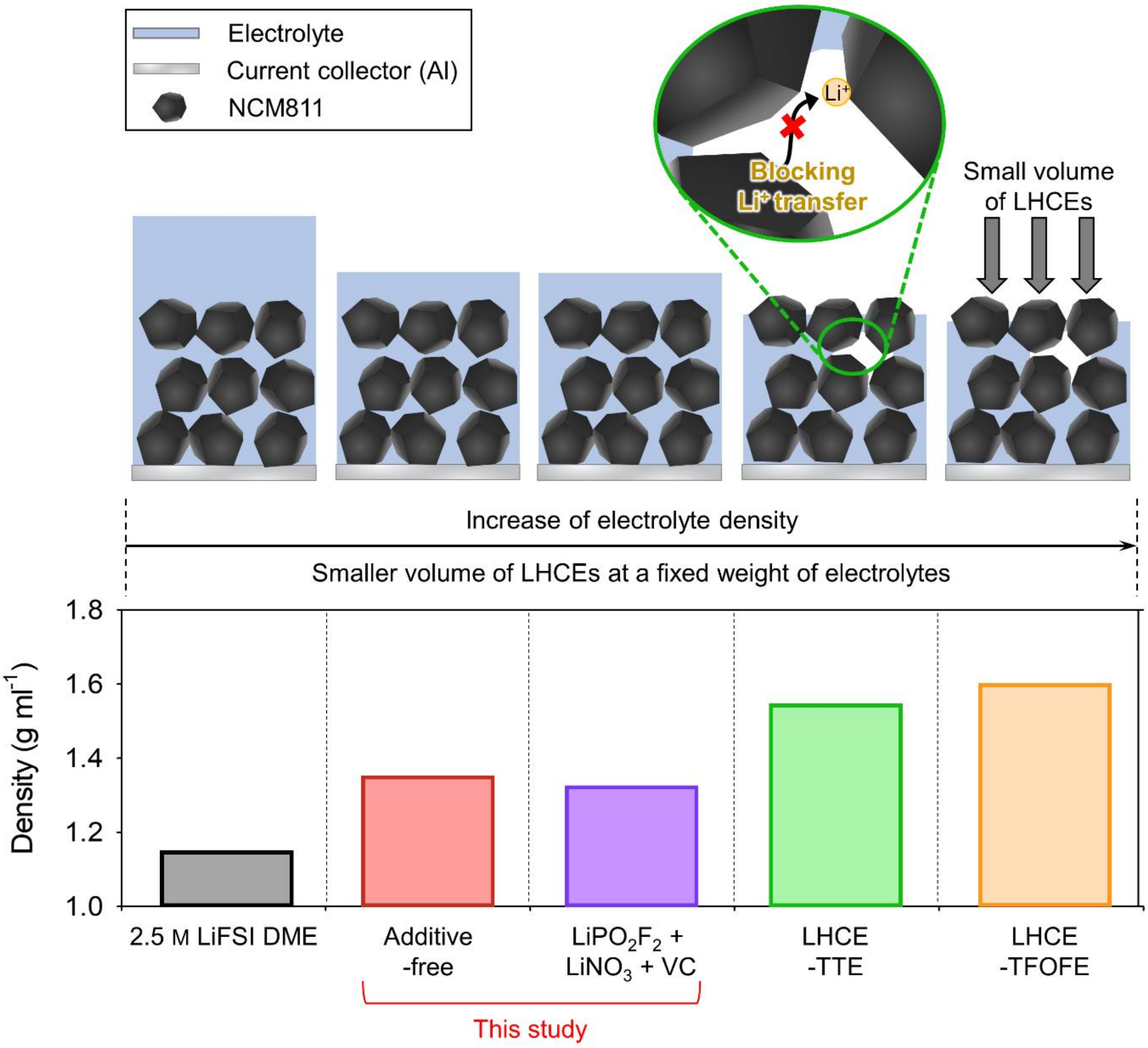

Figure S3. Schematic illustration showing different wetting states of the cathodes containing electrolytes with different densities.

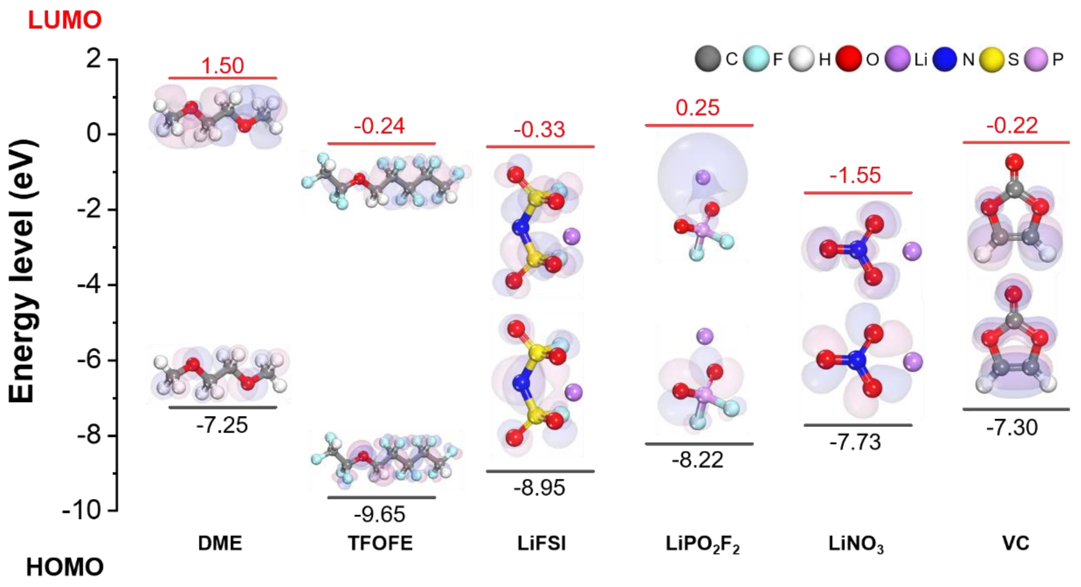

Figure S4. Energy-level diagram showing the HOMO and LUMO of the solvents (DME, TFOFE), salts (LiFSI, $LiPO_2F_2$, and $LiNO_3$), and additive (VC) used in this study. The isovalue of the orbital is 0.03 e/Å^3.

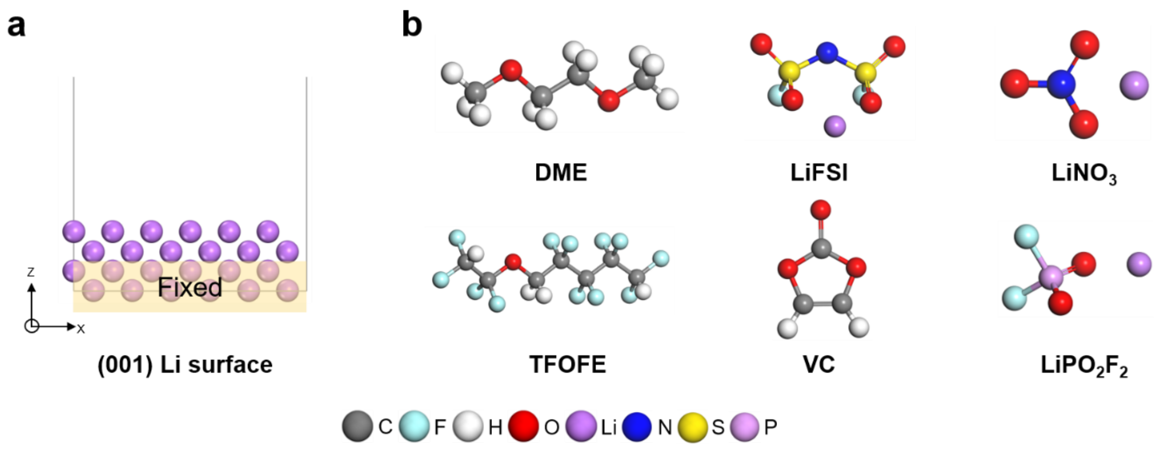

Figure S5. Model system and molecules used in DFT calculations. a) Li (001)-surface-slab model in which the bottom two fixed layers represent the bulk phase. b) DME, LiFSI, $LiNO_3$, TFOFE, VC, and $LiPO_2F_2$ molecules.

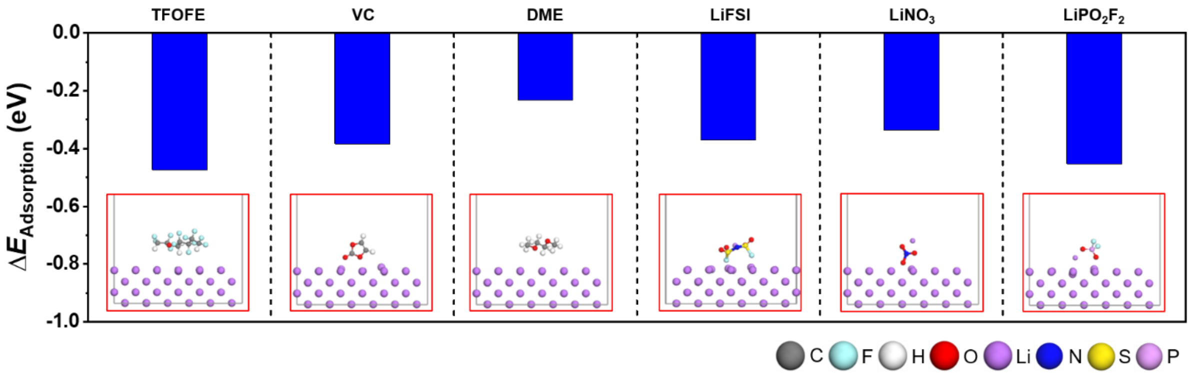

Figure S6. Adsorption energy ($\Delta E_{\text{Adsorption}}$) of TFOFE, VC, DME, LiFSI, $LiNO_3$, and $LiPO_2F_2$ on the (001) surface of Li metal. The most stable (optimized) systems are shown in red boxes.

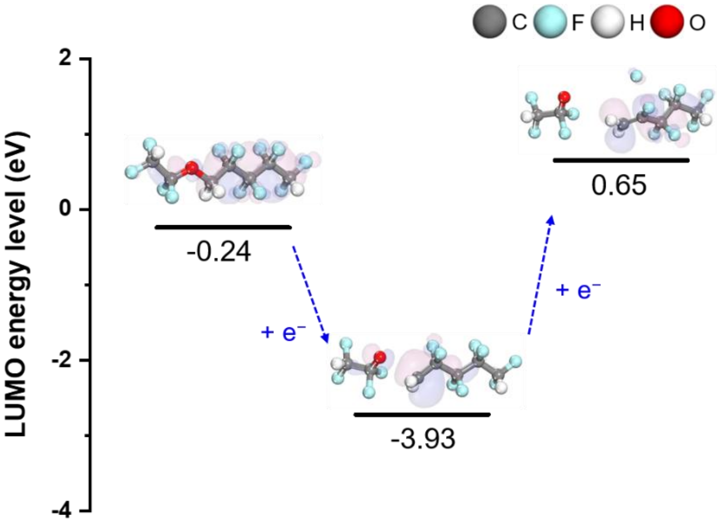

Figure S7. LUMO energy levels of TFOFE during one-, and two-electron reduction. The isovalue of the orbital is 0.03 *e*/Å^3.

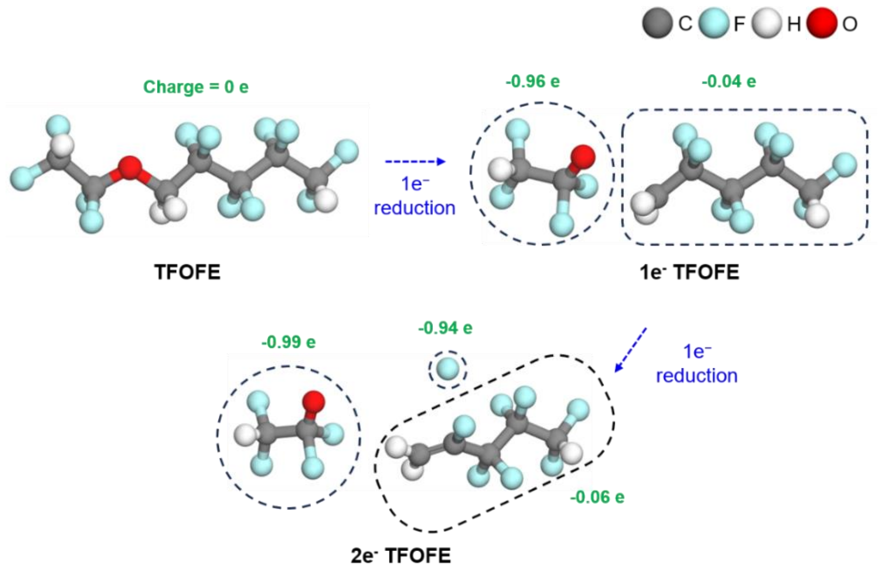

Figure S8. DFT optimized structure of TFOFE after one- and two-electron reduction. The charge dispersed on the dissociated molecules is written in green.

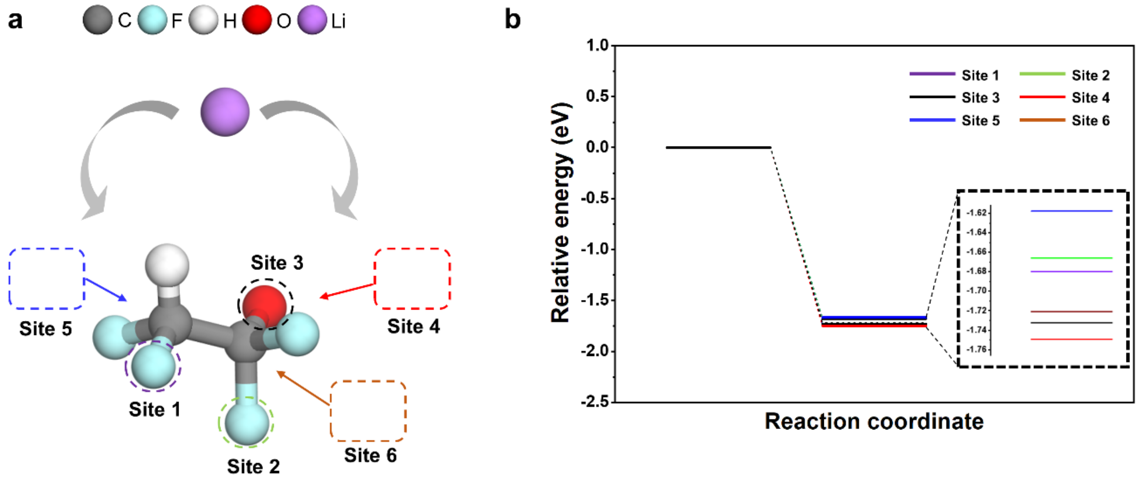

Figure S9. a) Adsorption sites of Li^+ on $C_2HOF_4^-$, marked by colored-dash boxes. b) Reaction-energy diagram of the adsorption of Li^+ on the different adsorption sites of $C_2HOF_4^-$.

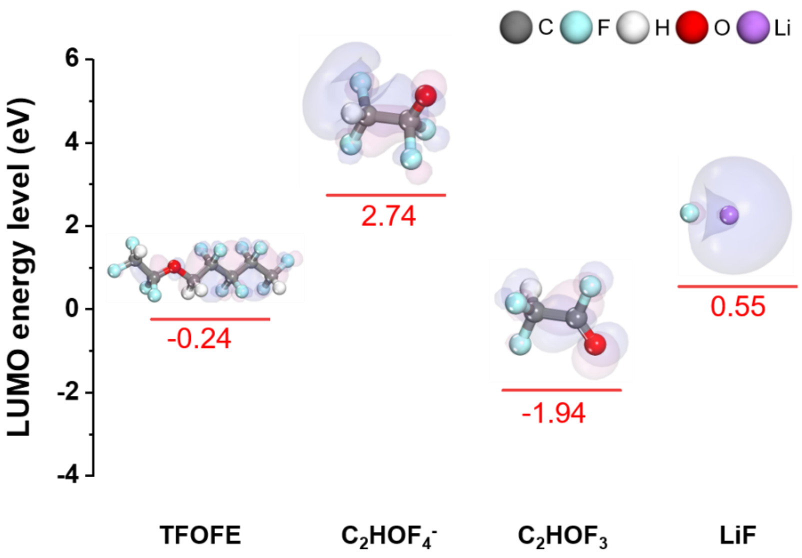

Figure S10. LUMO energy levels of TFOFE, $C_2HOF_4^-$, C_2HOF_3, and LiF. The isovalue of the orbital is 0.03 e/Å^3.

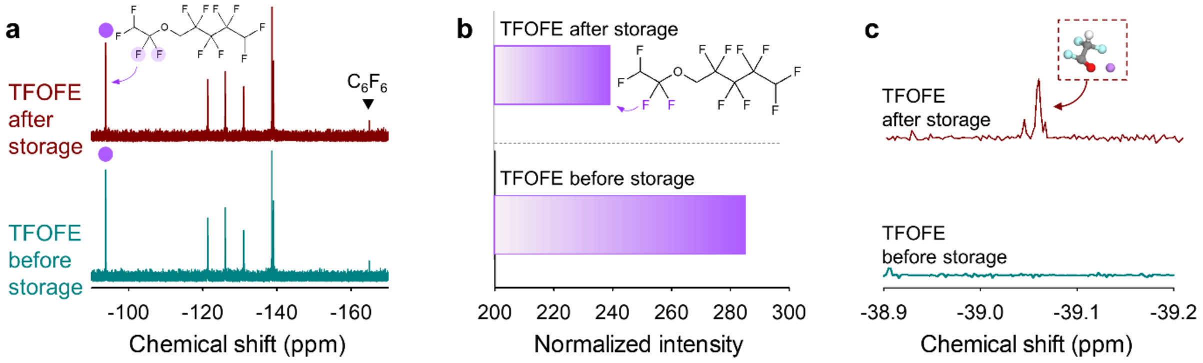

Figure S11. (a) ^{19}F NMR spectra of the TFOFE solvent before and after storage with Li-metal. (b) Integrated areas the peak corresponding to the F-functional group (see the purple-colored F atoms) in TFOFE for identifying the reductive decomposition of TFOFE, normalized using an internal reference (C_6F_6) of (a). (c) ^{19}F NMR spectra of the TFOFE solvent before and after storage with Li-metal (different region with (a)).

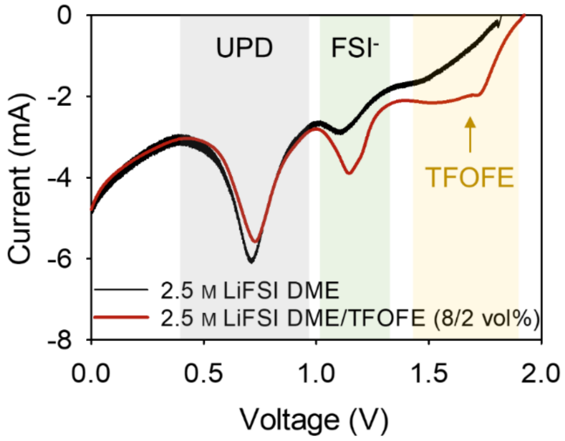

Figure S12. Electrochemical reduction behaviors of 2.5 M LiFSI DME and 2.5 M LiFSI DME/TFOFE (8/2 vol%) on the Li-metal of the Li||Cu cells; the results are measured at a scan rate of 0.1 mV/s during the initial Li plating. UPD represents "under potential decomposition."

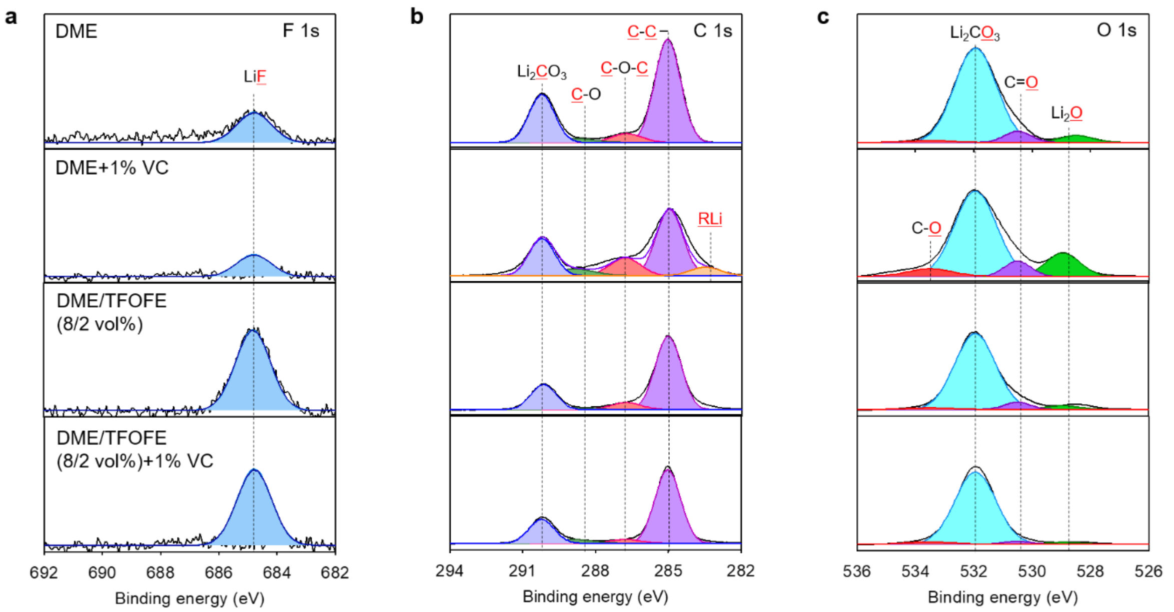

Figure S13. a) F 1s, b) C 1s, and c) O 1s XPS spectra of Li-metal anodes stored in solvents with and without 1% of VC (an additive) for 7 days to investigate the composition of the SEI layer formed in different electrolytes.

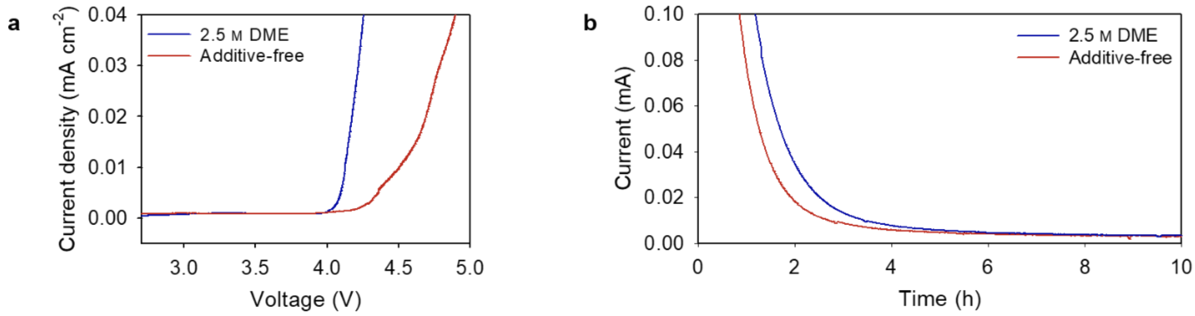

Figure S14. Comparison of the oxidation stability of the additive-free electrolyte and DME containing LiFSI (2.5 M) using a) LSV with a stainless-steel working electrode at a scan rate of 1 mV s^{-1} and b) electrochemical floating tests of Li||NCM811 full cells at 4.2 V vs. Li/Li^+

after precycling in 25 °C. The additive-free electrolyte comprises LiFSI (2.5 M) in DME/TFOFE (8/2 vol%).

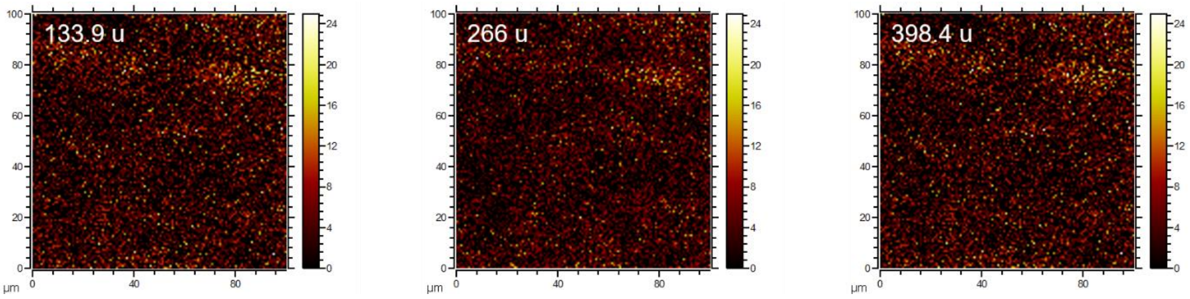

Figure S15. TOF–SIMS chemical maps (133.9, 266, and 398.4 au) of an Li-metal anode in the $LiPO_2F_2$ + $LiNO_3$ + VC electrolyte on 300 s of sputtering after precycling of Li||NCM811 full cells.

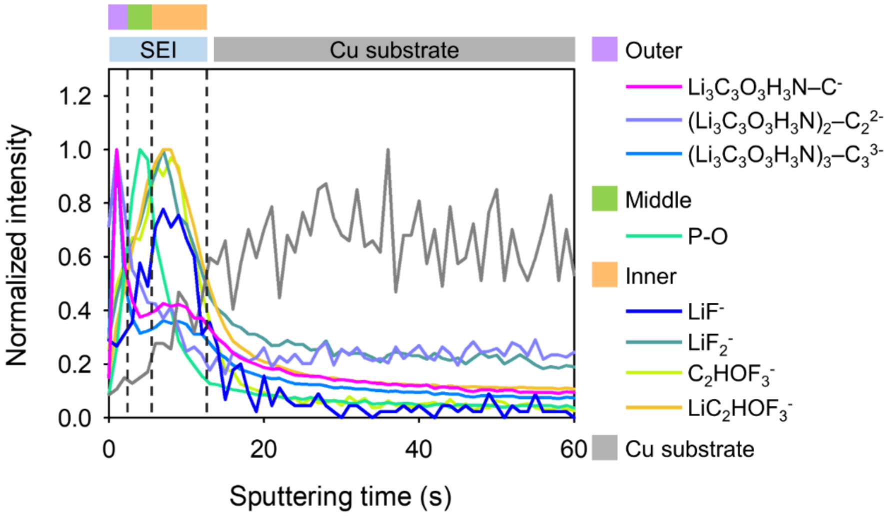

Figure S16. TOF–SIMS depth profile of the multilayered SEI on the Li-metal anode, containing the $LiPO_2F_2$ + $LiNO_3$ + VC electrolyte, after precycling of the Li||NCM811 full cells.

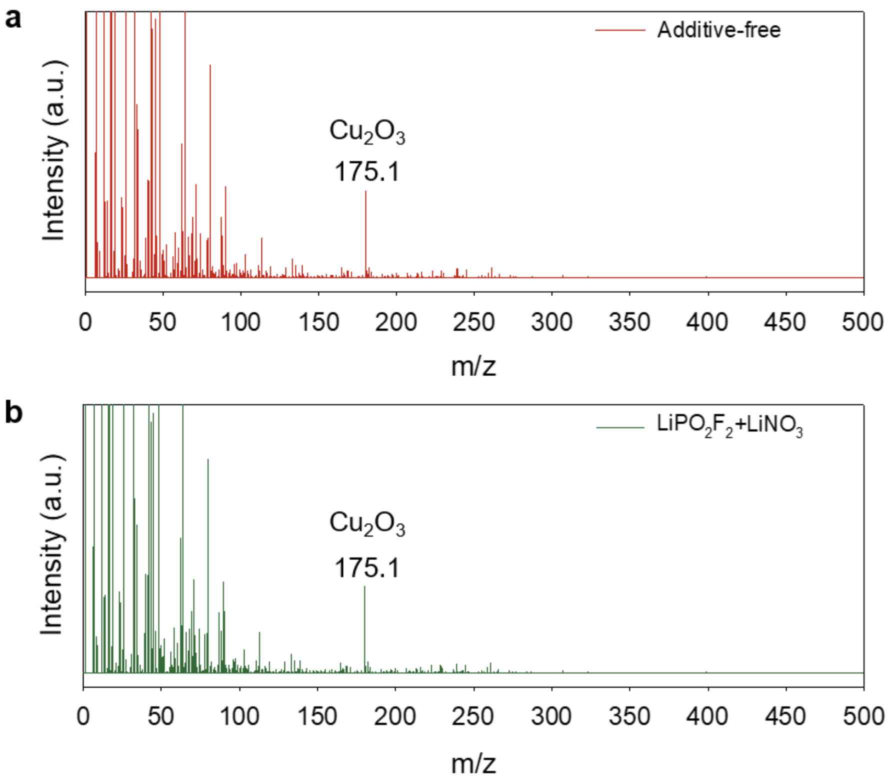

Figure S17. TOF–SIMS mass spectra of the a) additive-free and b) $LiPO_2F_2$ + $LiNO_3$ electrolyte. The additive-free electrolyte comprises LiFSI (2.5 M) in DME/TFOFE (8/2 vol%).

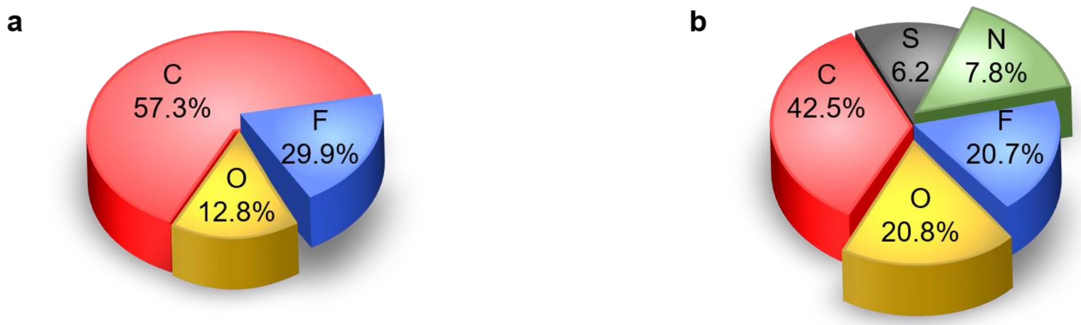

Figure S18. Atomic percentages of different elements in the a) inner and b) outer SEI layers of the multilayer SEI formed in the $LiPO_2F_2$ + $LiNO_3$ + VC electrolyte during precycling.

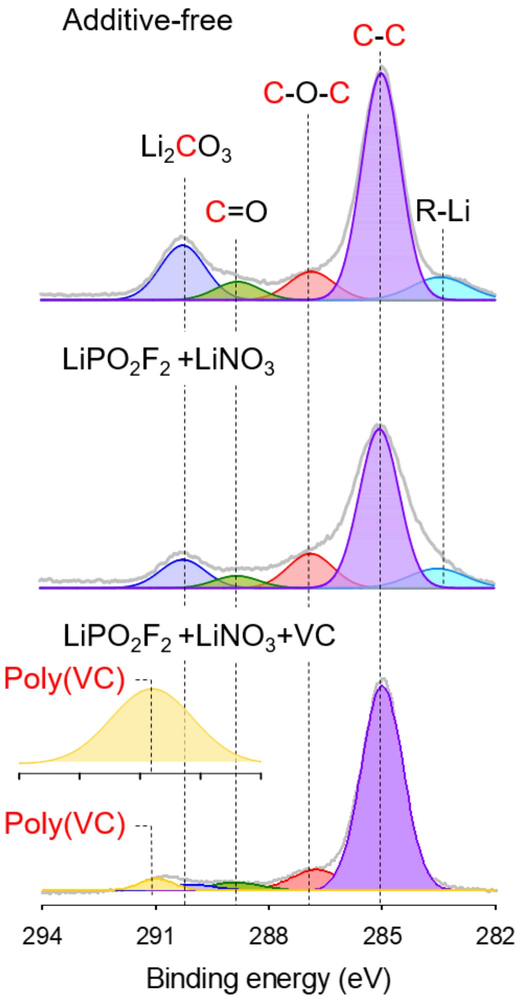

Figure S19. C 1s XPS results of Li-metal anodes precycled in the additive-free, $LiPO_2F_2$ + $LiNO_3$, and $LiPO_2F_2$ + $LiNO_3$ + VC electrolytes after precycling.

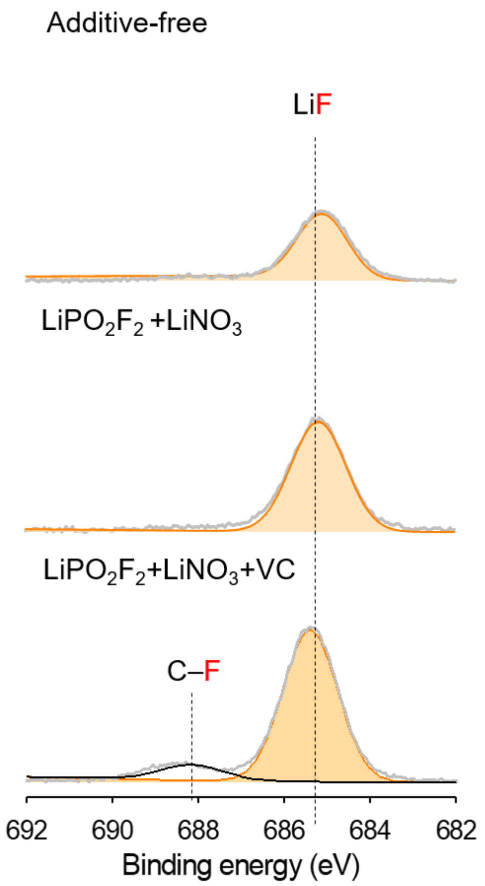

Figure S20. F 1s XPS spectra of Li-metal anodes precycled in the additive-free, $LiPO_2F_2$ + $LiNO_3$, and $LiPO_2F_2$ + $LiNO_3$ + VC electrolytes.

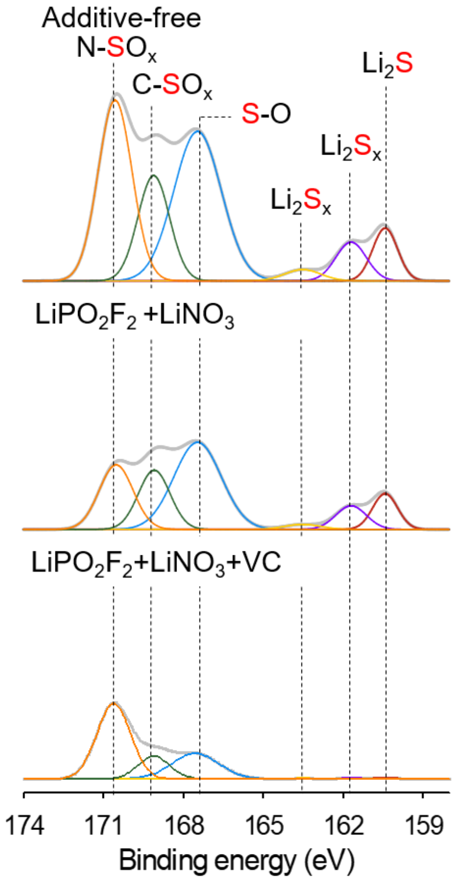

Figure S21. S 2p XPS spectra of Li-metal anodes precycled in the additive-free, $LiPO_2F_2$ + $LiNO_3$, and $LiPO_2F_2$ + $LiNO_3$ + VC electrolytes.

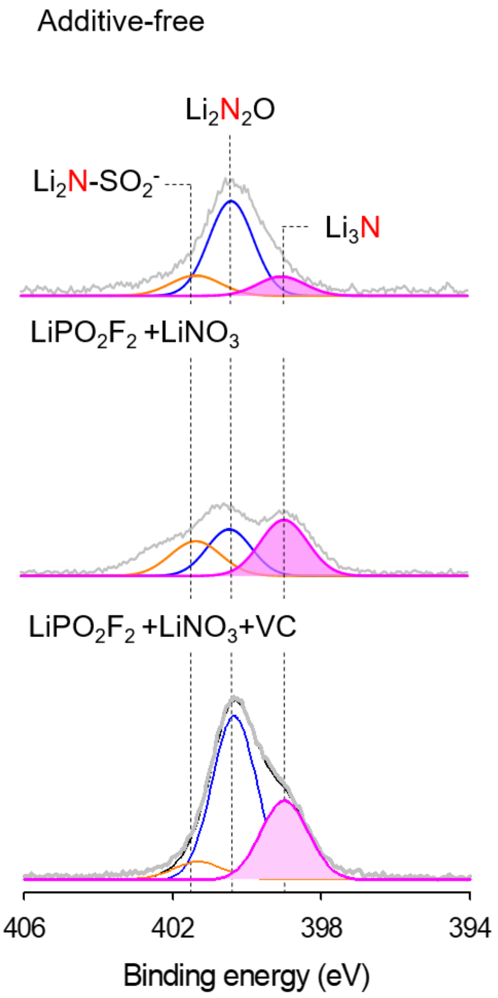

Figure S22. N 1s XPS spectra of Li-metal anodes precycled in the additive-free, $LiPO_2F_2$ + $LiNO_3$, and $LiPO_2F_2$ + $LiNO_3$ + VC electrolytes.

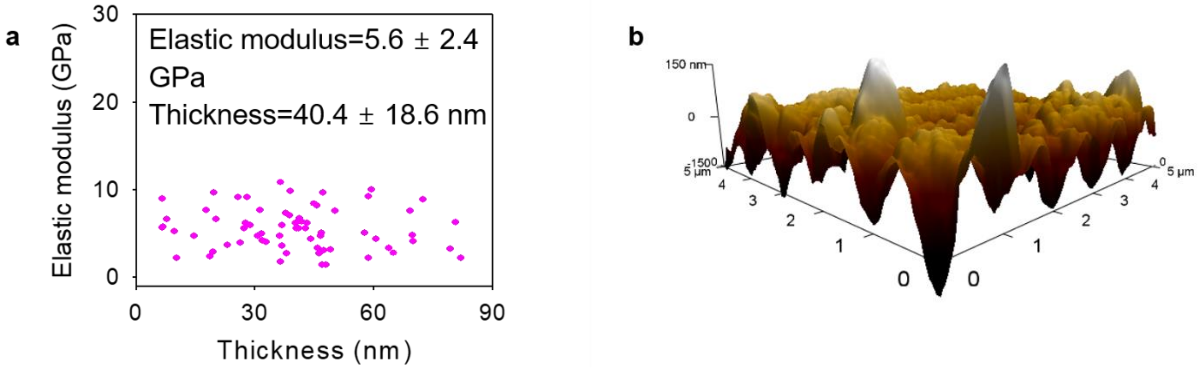

Figure S23. a) Elastic modulus and thickness of the SEI layer formed in the $LiPO_2F_2$ + VC electrolyte. b) AFM 3D image recorded before the measurement of the construction of force–indentation depth curves of the SEI formed in the $LiPO_2F_2$ + VC electrolyte.

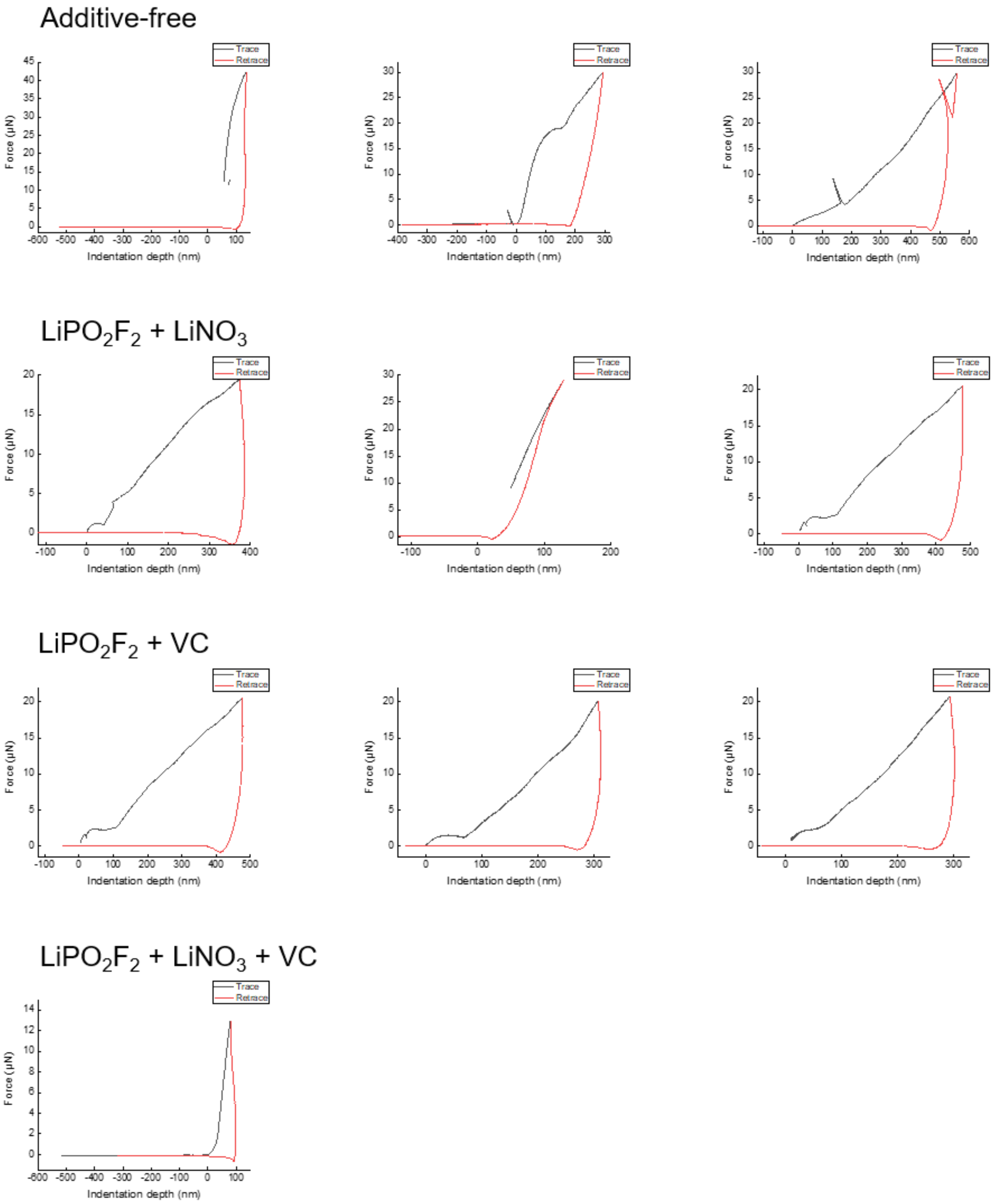

Figure S24. The excluded force–indentation depth curves of Li-metal anodes formed in the additive-free, $LiPO_2F_2$ + VC, and $LiPO_2F_2$ + $LiNO_3$ + VC electrolytes on precycling.

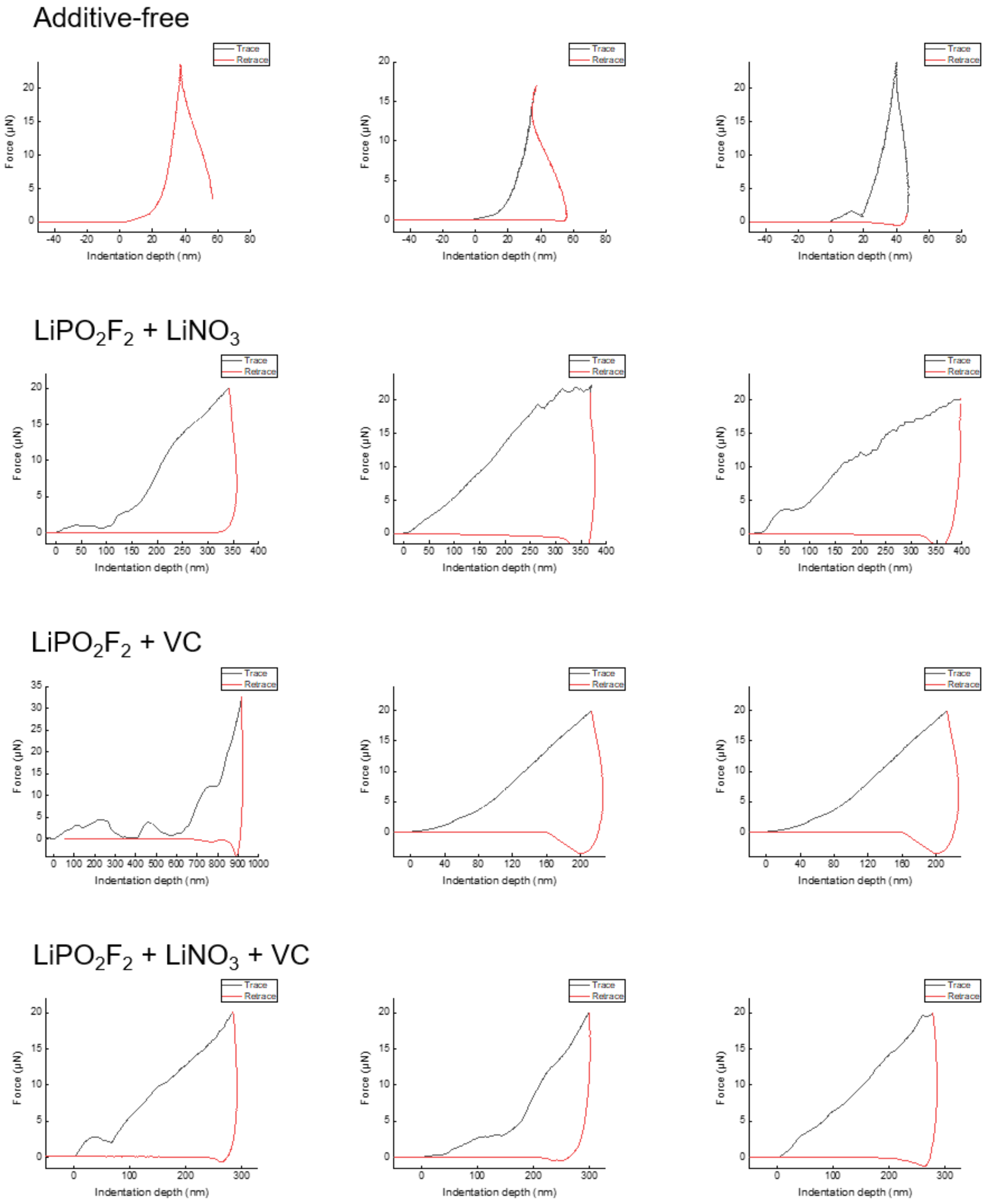

Figure S25. The excluded force–indentation depth curves of Li-metal anodes after 1 cycle in the additive-free, $LiPO_2F_2$ + VC, and $LiPO_2F_2$ + $LiNO_3$ + VC electrolytes.

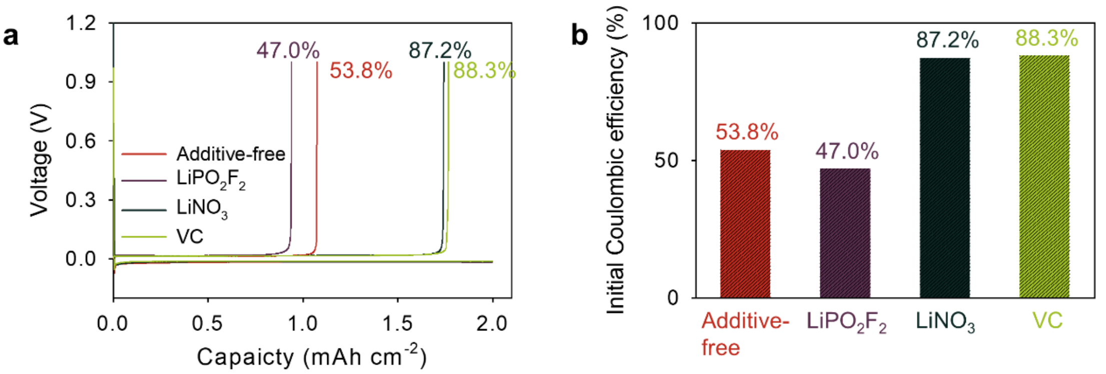

Figure S26. Electrochemical performance of Li||Cu cells containing the additive-free, $LiPO_2F_2$ + VC, and $LiPO_2F_2$ + $LiNO_3$ + VC electrolytes. a) Voltage profiles of initial Li plating/stripping in the Li||Cu cells. b) Coulombic efficiency during initial Li plating/stripping in the Li||Cu cells.

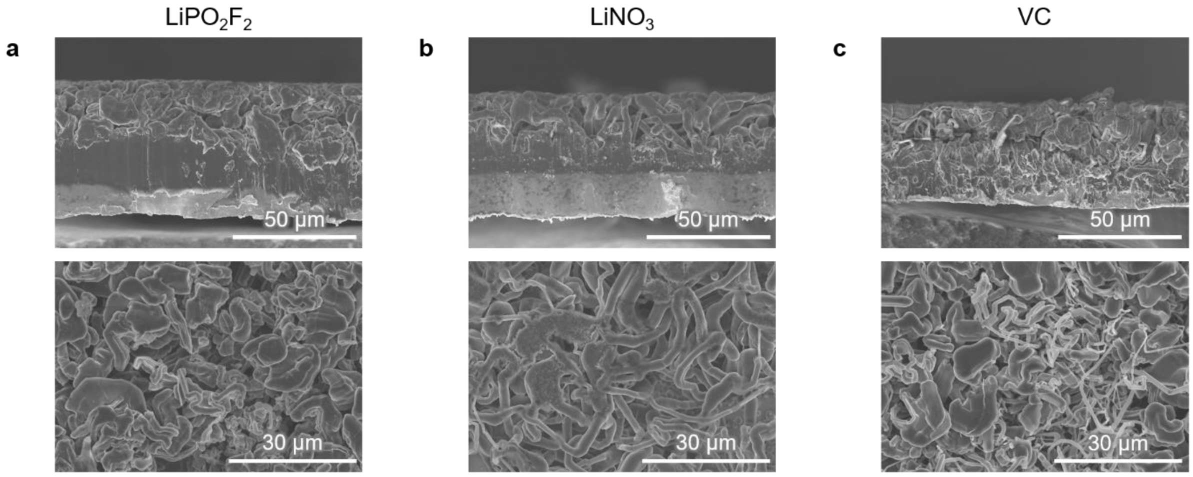

Figure S27. Cross-sectional and surface SEM images of Li-metal anodes extracted from Li||Cu cells containing the a) $LiPO_2F_2$, b) $LiNO_3$, and c) VC electrolytes after initial Li plating at 1mA cm^{-2} and 2 mAh cm^{-2}.

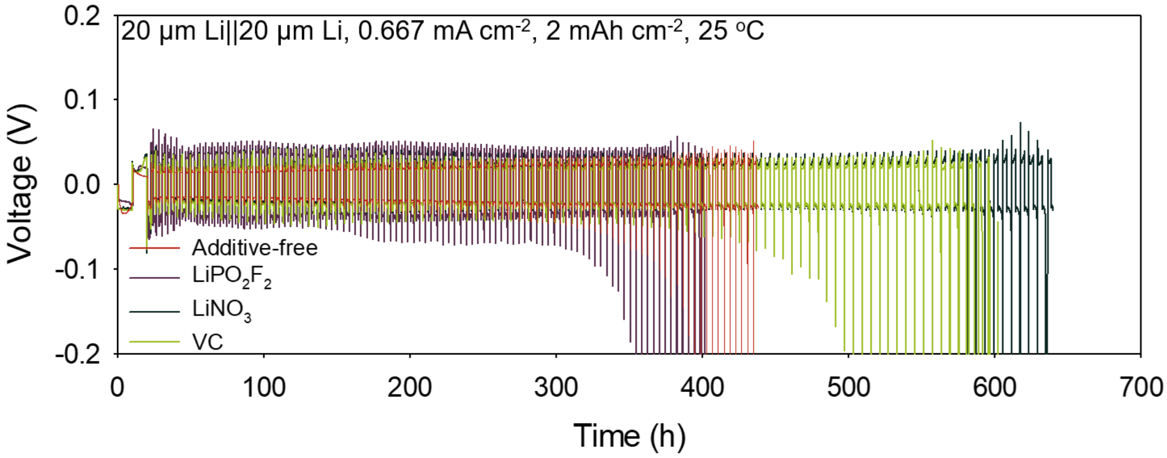

Figure S28. Voltage profiles of cycling Li||Li cells containing the additive-free, $LiPO_2F_2$, $LiNO_3$, and VC electrolytes at 0.667 mA cm^{-2} and 2 mAh cm^{-2}.

In the figure, additive-free indicates the additive-free electrolyte comprising LiFSI (2.5 M) in DME/TFOFE (8/2 vol%); $LiPO_2F_2$ indicates a solution comprising LiFSI (2.5 M) and 0.3 wt.% of $LiPO_2F_2$ in DME/TFOFE (8/2 vol%); $LiNO_3$ indicates a solution comprising LiFSI (2.5 M) and 1 wt.% of $LiNO_3$ in DME/TFOFE (8/2 vol%); and VC indicates a solution comprising LiFSI (2.5 M) and 1 wt.% of VC in DME/TFOFE (8/2 vol%).

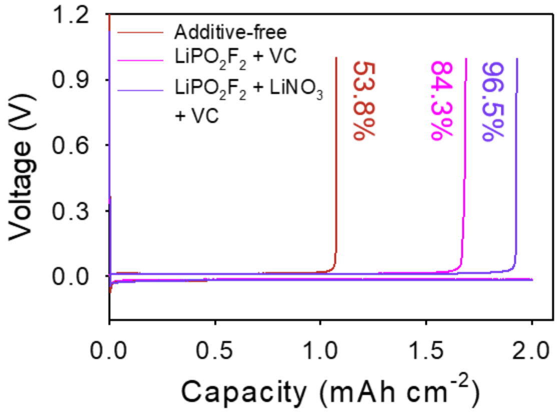

Figure S29. Voltage profiles of initial Li plating/stripping in Li||Cu cells containing the additive-free, $LiPO_2F_2$ + VC, and $LiPO_2F_2$ + $LiNO_3$ + VC electrolytes.

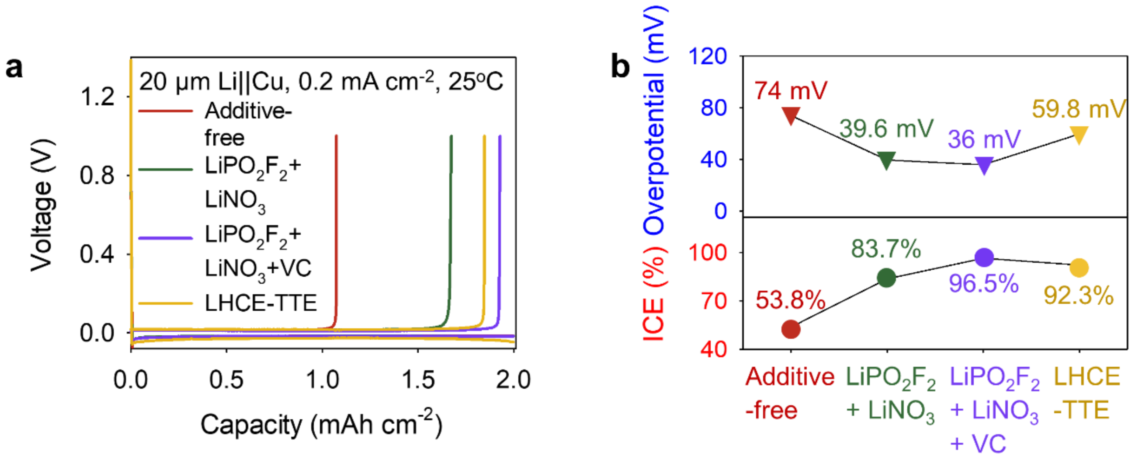

Figure S30. a) Voltage curves for initial Li plating and stripping of the Li||Cu cells with different electrolytes. b) ICE and nucleation overpotentials for initial Li plating on a Cu substrate in the Li||Cu cells.

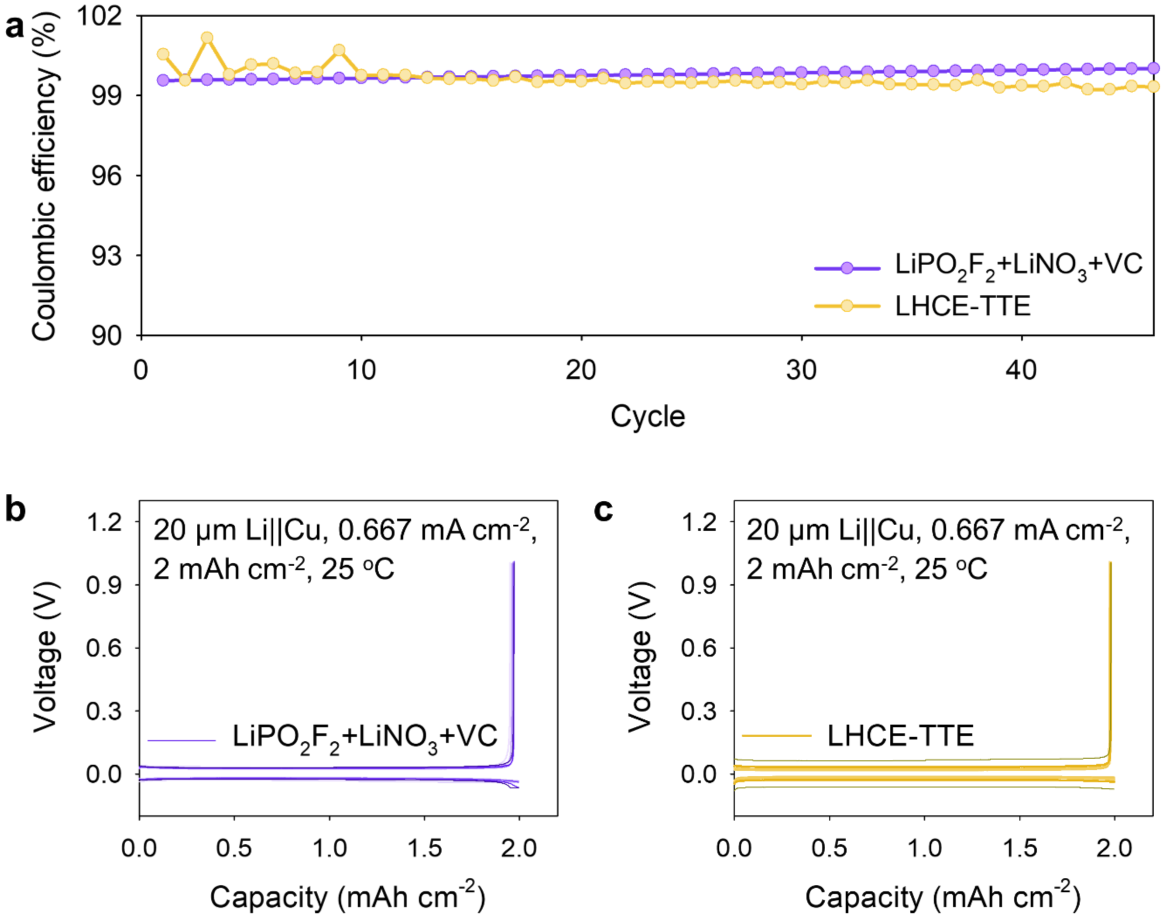

Figure S31. a) CEs of the Li||Cu cells with $LiPO_2F_2$ + $LiNO_3$ + VC and LHCE–TTE electrolytes. Voltage curves of the Li||Cu cells with b) $LiPO_2F_2$ + $LiNO_3$ + VC and c) LHCE–TTE electrolytes during 25 cycles at 25 ℃.

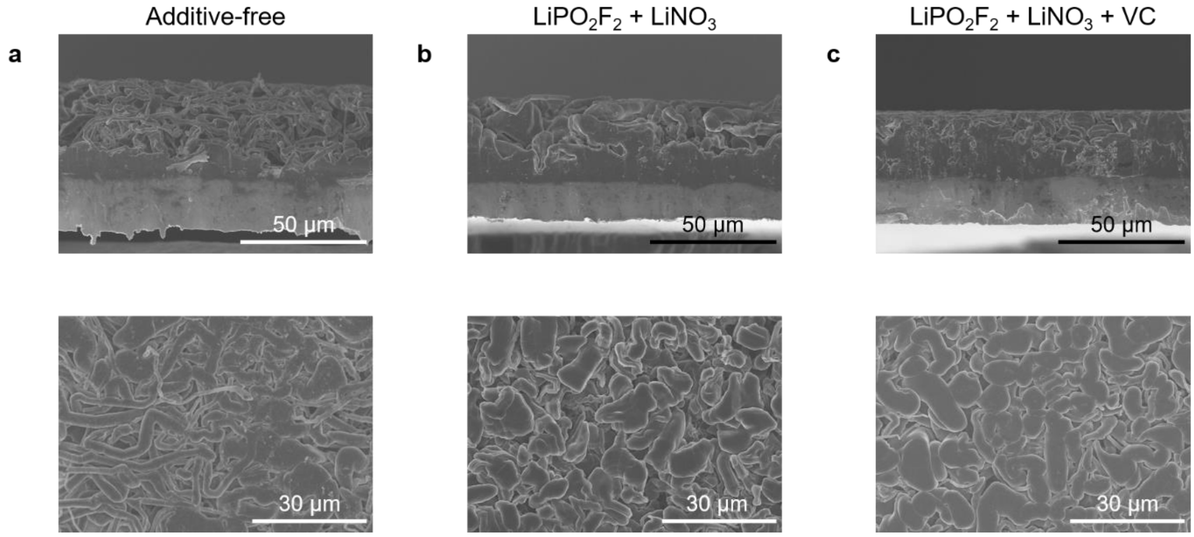

Figure S32. Cross-sectional and surface SEM images of an Li-metal electrode extracted from Li||Cu cells containing the a) additive-free, b) $LiPO_2F_2$ + $LiNO_3$, and c) $LiPO_2F_2$ + $LiNO_3$ + VC electrolytes after initial Li plating at 1 mA cm^{-2} and 2 mAh cm^{-2}.

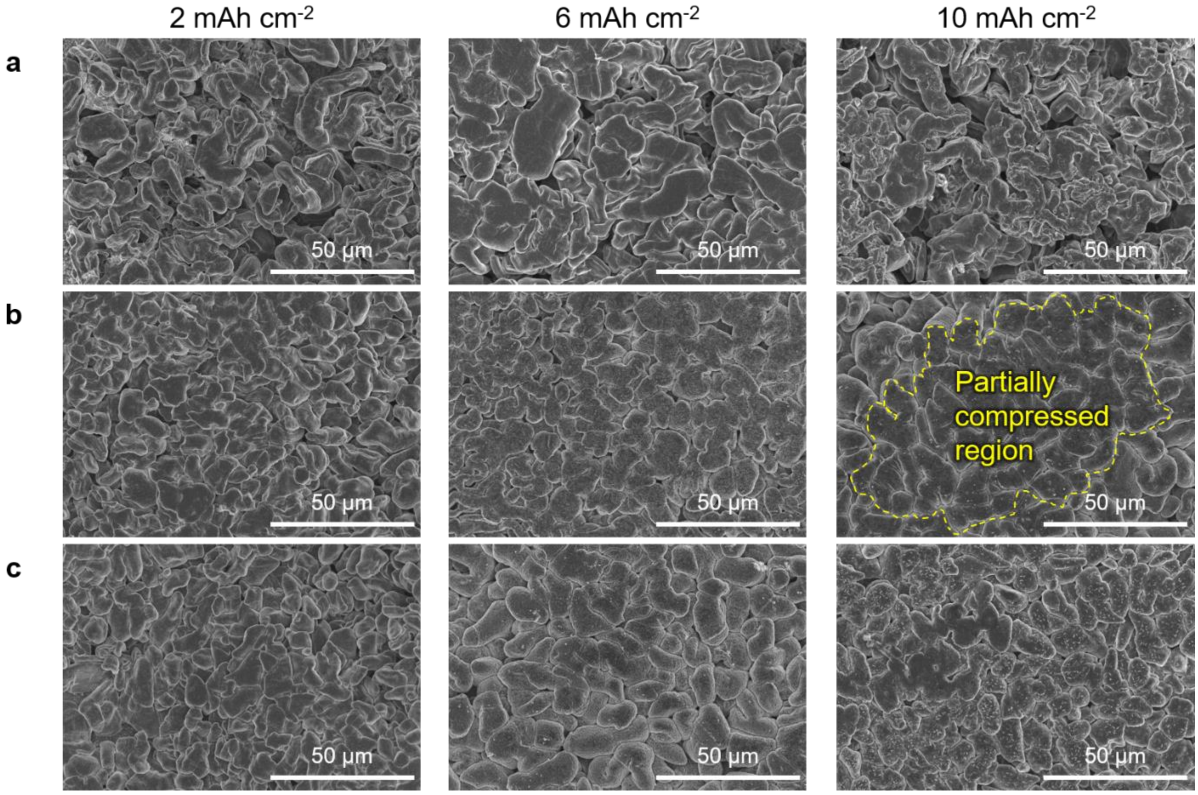

Figure S33. Surface SEM images of an Li-metal electrode extracted from Li||Cu cells containing the a) additive-free, b) $LiPO_2F_2$ + $LiNO_3$, and c) $LiPO_2F_2$ + $LiNO_3$ + VC electrolyte after initial Li plating at different areal capacities.

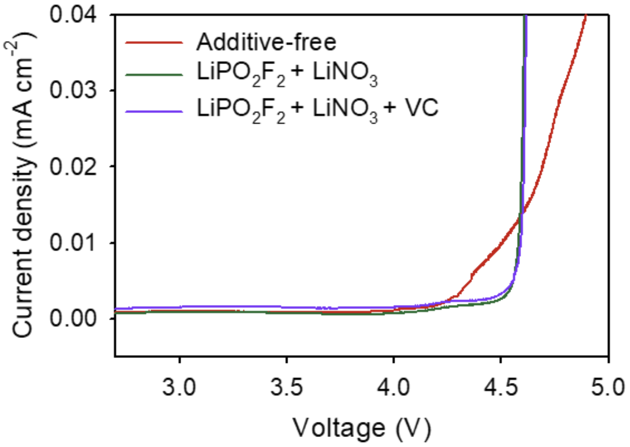

Figure S34. LSV of electrolytes with a stainless-steel working electrode at a scan rate of 1 mV s^{-1} and 25 ℃.

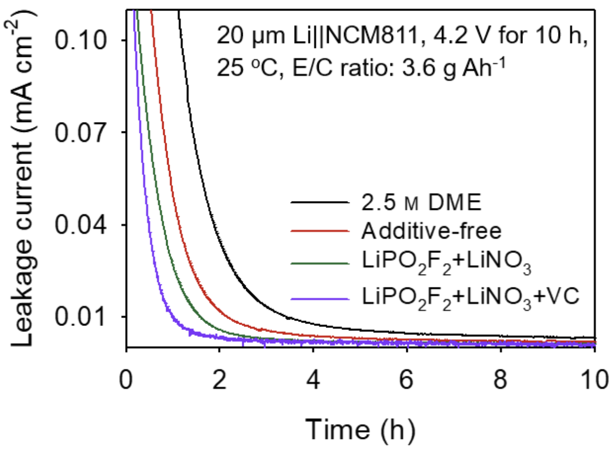

Figure S35. Electrochemical floating test of Li||NCM811 full cells at 4.2 V vs. Li/Li$^+$ after precycling in different electrolytes.

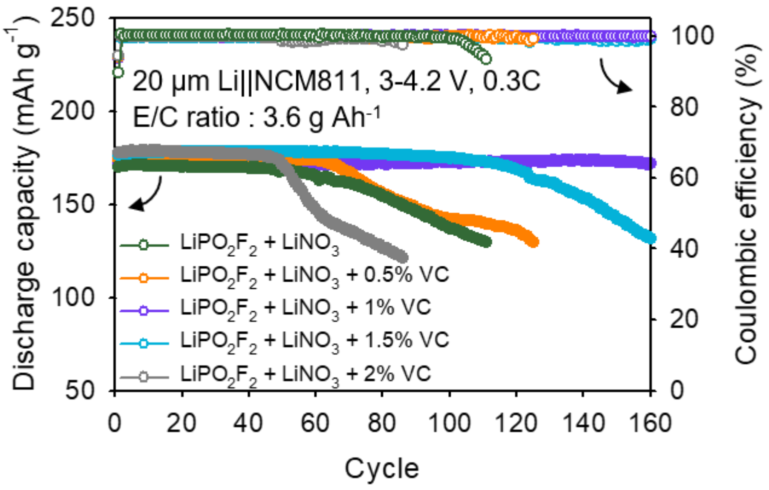

Figure S36. Cycling performance of Li||NCM811 full cells containing electrolytes with different concentrations of the additive VC in the voltage range of 3.0–4.2 V.

The $LiPO_2F_2$ + $LiNO_3$ electrolyte comprises LiFSI (2.5 M), 0.3 wt.% of $LiPO_2F_2$, and 1 wt.% of $LiNO_3$ in DME/TFOFE (8/2 vol%).

The $LiPO_2F_2$ + $LiNO_3$ + 0.5% VC electrolyte comprises LiFSI (2.5 M), 0.3 wt.% of $LiPO_2F_2$, 1 wt.% of $LiNO_3$, and 0.5 wt.% of VC in DME/TFOFE (8/2 vol%).

The $LiPO_2F_2$ + $LiNO_3$ + 1% VC electrolyte comprises LiFSI (2.5 M), 0.3 wt.% of $LiPO_2F_2$, 1 wt.% of $LiNO_3$, and 1 wt.% of VC in DME/TFOFE (8/2 vol%).

The $LiPO_2F_2$ + $LiNO_3$ + 1.5% VC electrolyte comprises LiFSI (2.5 M), 0.3 wt.% of $LiPO_2F_2$, 1 wt.% of $LiNO_3$, and 1.5 wt.% of VC in DME/TFOFE (8/2 vol%).

The $LiPO_2F_2$ + $LiNO_3$ + 2% VC electrolyte comprises LiFSI (2.5 M), 0.3 wt.% of $LiPO_2F_2$, 1 wt.% of $LiNO_3$, and 2 wt.% of VC in DME/TFOFE (8/2 vol%).

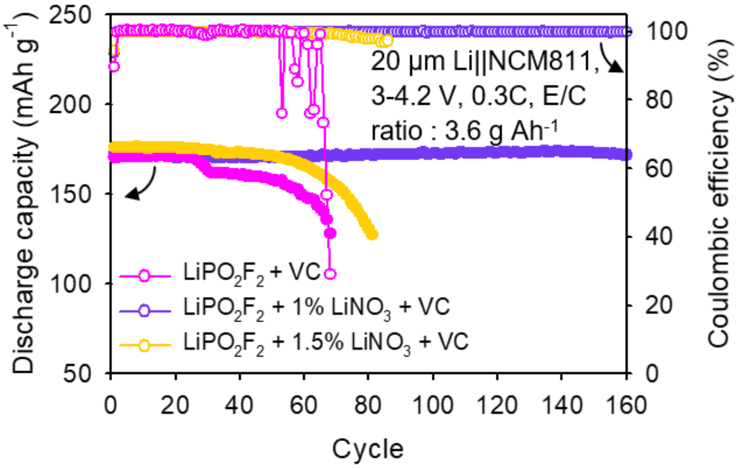

Figure S37. Cycling performance of Li||NCM811 full cells with electrolytes containing different concentrations of the additive $LiNO_3$.

The $LiPO_2F_2$ + VC electrolyte comprises LiFSI (2.5 M), 0.3 wt.% of $LiPO_2F_2$, and 1 wt.% of VC in DME/TFOFE (8/2 vol%)

The $LiPO_2F_2$ + 1% $LiNO_3$ + VC electrolyte comprises LiFSI (2.5 M), 0.3 wt.% of $LiPO_2F_2$, 1 wt.% of $LiNO_3$, and 1 wt.% of VC in DME/TFOFE (8/2 vol%)

The $LiPO_2F_2$ + 1.5% $LiNO_3$ + VC electrolyte comprises LiFSI (2.5 M), 0.3 wt.% $LiPO_2F_2$, 1.5 wt.% of $LiNO_3$, and 1 wt.% of VC in DME/TFOFE (8/2 vol%)

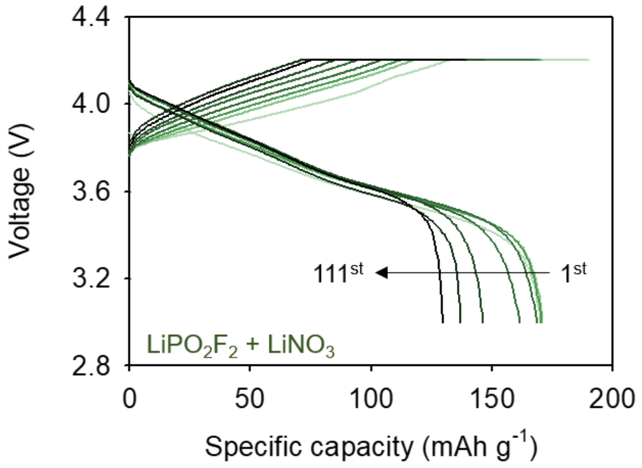

Figure S38. Voltage profiles of Li||NCM811 full cells cycled in the $LiPO_2F_2$ + $LiNO_3$ electrolyte.

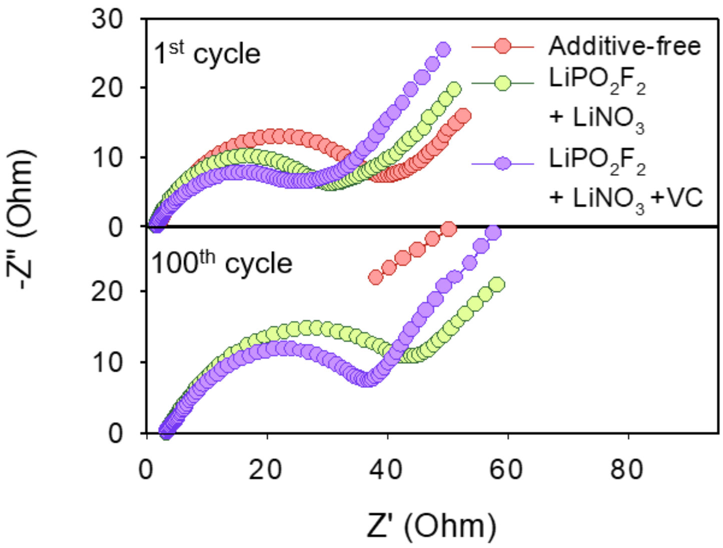

Figure S39. EIS results of Li||NCM811 full cells containing different electrolytes after 1 cycle and 100 cycles.

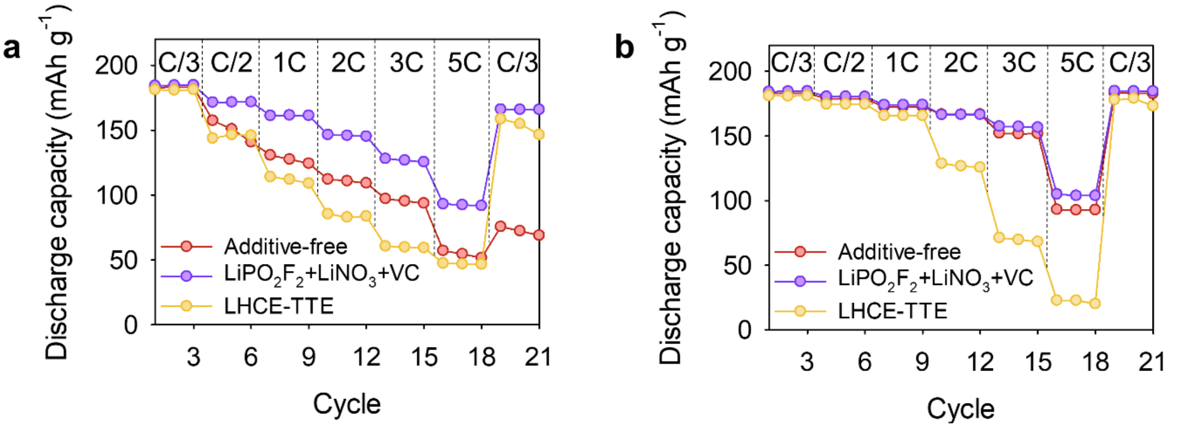

Figure S40. Rate performances of the Li||NCM811 full cells at a) various charge rates and a constant discharge rate of C/3 and b) various discharge rates and a fixed charge rate of C/3.

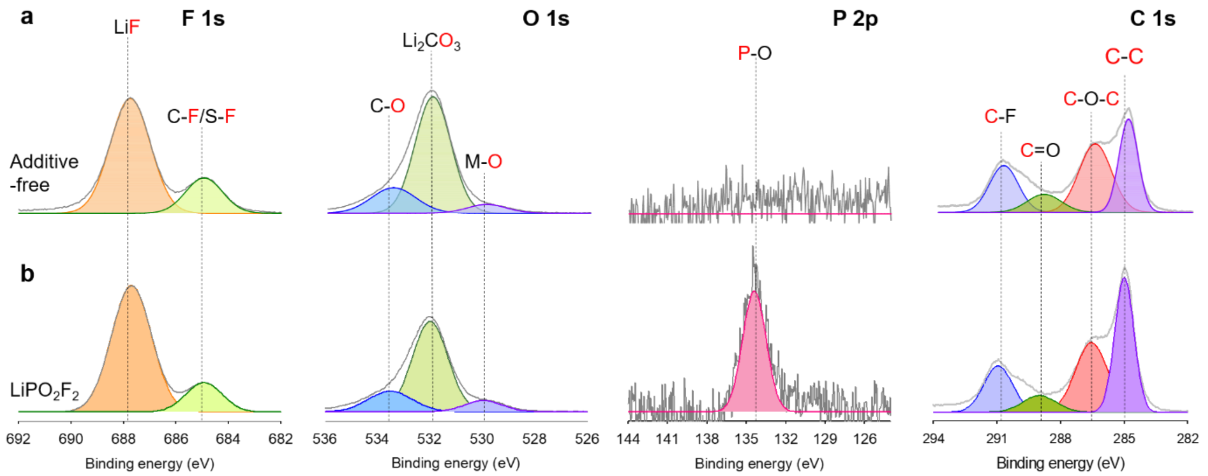

Figure S41. F 1s, O 1s, P 2p, and C 1s XPS patterns of NCM811 cathodes cycled in the a) additive-free and b) $LiPO_2F_2$ electrolyte.

The additive-free electrolyte comprises LiFSI (2.5 M) in DME/TFOFE (8/2 vol%)

$LiPO_2F_2$ comprises LiFSI (2.5 M) and 0.3 wt.% of $LiPO_2F_2$ in DME/TFOFE (8/2 vol%)

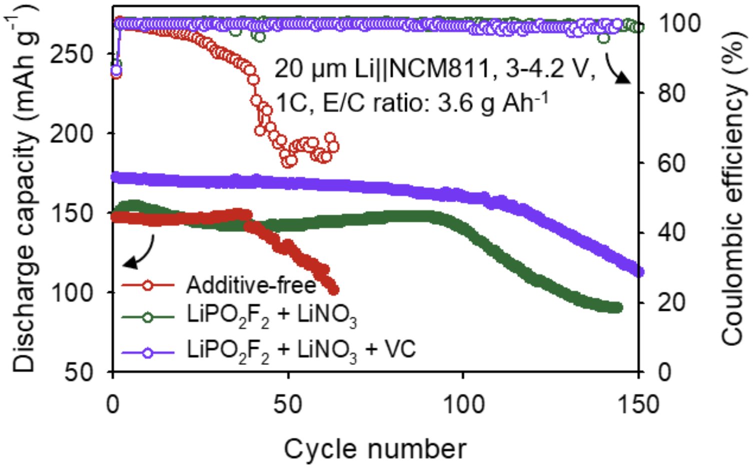

Figure S42. Cycling performance of Li||NCM811 full cells containing different electrolytes at 1C.

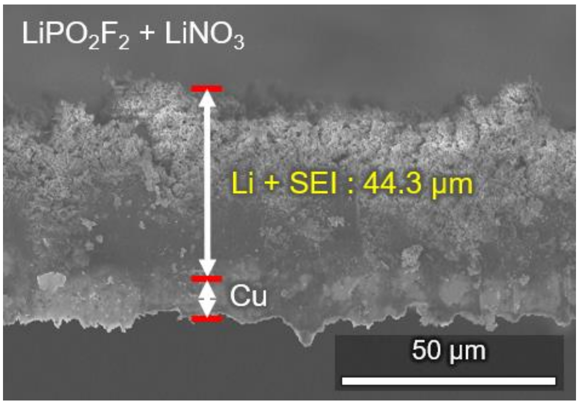

Figure S43. Cross-sectional SEM image of an Li-metal anode extracted from an Li||NCM811 full cell containing the $LiPO_2F_2$ + $LiNO_3$ electrolyte after 50 cycles.

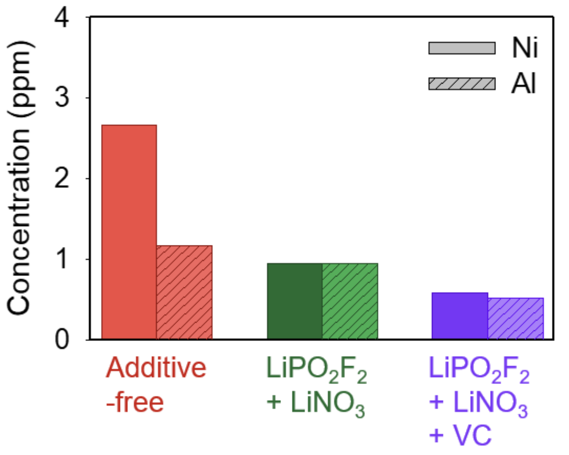

Figure S44. ICP–OES analysis of Ni and Al dissolution from NCM811 cathodes extracted from Li||NCM811 full cells in the fully charged state.

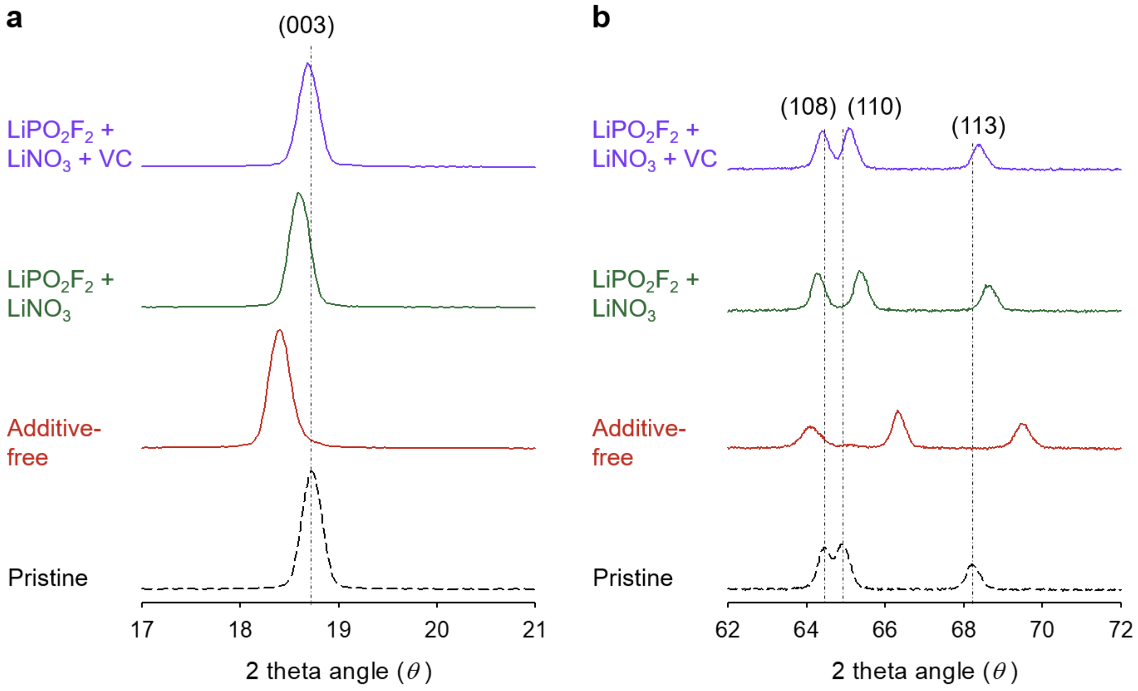

Figure S45. XRD analyses of NCM811 cathodes extracted from Li||NCM811 full cells containing different electrolytes after 100 cycles.
